# Construction of Large‐Scale Bioengineered Hair Germs and In Vivo Transplantation

**DOI:** 10.1002/advs.202416361

**Published:** 2025-03-05

**Authors:** Yangpeng Chen, Yuhui Hou, Jiejian Chen, Jiaojiao Bai, Lijuan Du, Chen Qiu, Hanzhou Qi, Xuanbei Liu, Junfei Huang

**Affiliations:** ^1^ Department of Plastic and Aesthetic Surgery Department of Hematology Nanfang Hospital Southern Medical University Guangzhou Guangdong 510515 China; ^2^ Department of Medical Oncology Guangzhou First People's Hospital Guangzhou Medical University Guangdong 510180 China; ^3^ Department of Haematology Guangdong Provincial People's Hospital (Guangdong Academy of Medical Sciences) Guangzhou Guangdong 510030 China; ^4^ Department of Oncology Shanghai General Hospital 650 Xinsongjiang Road Songjiang District Shanghai 201620 China

**Keywords:** bioengineered hair germ, hair regeneration, microfluidics, mucopolysaccharide, thermodynamic incompatibility

## Abstract

Hair follicle (HF) regeneration technology holds promise for treating hair loss, but creating a biomimetic structure that mimics the natural follicle microenvironment remains challenging. Here a novel bioengineered hair germ (BHG) is developed using thermodynamically incompatible mucopolysaccharides to enhance HF regeneration efficiency. Mucopolysaccharide‐based hydrogels are synthesized by grafting amino and diethylamino groups (dihydroxyphenylalanine‐grafted hyaluronic acid (HME) hydrogels) for rapid gelation and strong wetting adhesion. Dual‐layered microspheres are fabricated using a co‐flow microfluidic system, with HME as the outer shell and gelatin methacrylate (GelMA) as the core, achieving thermodynamic incompatibility. The Wnt3a protein is encapsulated for sustained release. RNA sequencing, reverse transcription quantitative polymerase chain reaction (RT‐qPCR), and functional validation are used to study the molecular mechanisms of HF regeneration. Results show that HME hydrogels exhibit excellent adhesion, shear‐thinning behavior, and biocompatibility. The microspheres release Wnt3a for up to 9 days, with high‐throughput sequencing revealing upregulation of HF regeneration genes like *Ctnnb1* and *Lef1*, and activation of the Wnt signaling pathway, while hypoxia‐related genes such as *Hif‐1ɑ* are downregulated. Pathway enrichment analyses confirm the enrichment of HF regeneration pathways. In conclusion, the HME‐based BHG microspheres effectively promote in vivo HF regeneration, offering a promising solution for hair loss treatment and regeneration.

## Graphical abstract

Here, a novel bioengineered hair germ (BHG) system is developed using thermodynamically incompatible mucopolysaccharides for efficient hair follicle regeneration. The BHG microspheres, produced via microfluidic technology, encapsulate Wnt3a for sustained release, enhancing mesenchymal stem cell differentiation and activating key regeneration pathways. In vivo experiments confirm the system's potential for scalable hair regeneration, offering a promising treatment for hair loss.

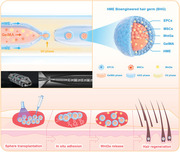

## 1 Introduction

Hair loss or alopecia is a common condition that affects millions of individuals worldwide, significantly affecting their quality of life and self‐esteem.^[^ ^1^ ^]^ Despite the availability of various treatment options including pharmaceutical therapies and hair transplantation, achieving natural and long‐lasting hair regeneration remains a challenge.^[^ ^2^, ^3^ ^]^ Hair follicle (HF) regeneration is a complex process involving the coordinated interaction of multiple cell types and signaling pathways, particularly the Wnt/β–catenin pathway, which plays a critical role in HF morphogenesis and cycling.^[^ ^4^, ^5^, ^6^, ^7^ ^]^

Recent advances in tissue engineering and regenerative medicine have provided new opportunities for the development of effective treatments. The concept of a bioengineered hair germ (BHG), which mimics the structure and function of natural HFs, has gained considerable attention.^[^ ^8^, ^9^ ^]^ By replicating the microenvironment of the HF niche, BHG systems can provide necessary cues for stem cell differentiation and HF regeneration. However, designing a functional BHG that meets the structural, mechanical, and biochemical requirements for HF regeneration remains challenging.

One significant challenge is the difficulty in obtaining sufficient quantities of viable mesenchymal cells (MSCs) and epithelial cells (EPCs) for effective regeneration. Traditional methods of isolating these cells often result in loss of their intrinsic properties during in vitro expansion, which can compromise their functionality when reintroduced into the host environment.^[^ ^10^, ^11^, ^12^ ^]^

Moreover, the epithelial–mesenchymal interactions that are crucial for HF morphogenesis and regeneration are complex and not fully understood.^[^ ^7^, ^13^ ^]^ Although studies have demonstrated that coculturing keratinocytes with dermal papilla cells (DPCs) can enhance HF formation, the technical hurdles associated with replicating these interactions in vitro remain a significant barrier.^[^ ^14^ ^]^ Additionally, the current biomaterials used in tissue engineering, although promising, often do not adequately mimic the natural extracellular matrix required for optimal cell behavior and HF development.^[^ ^15^, ^16^ ^]^

Another limitation is the scalability and reproducibility of tissue engineering processes. Microwell array chips, 3D printing, and other methods have been employed to facilitate the creation of bionic double‐layered structures.^[^ ^8^, ^17^ ^]^ However, these methods are labor‐intensive and not easily adaptable for large‐scale production, which is essential for clinical applications (Table S1, Supporting Information).

Moreover, the integration of advanced technologies, such as microfluidic devices, allows for better control over cellular organization and interactions within these constructs.^[^ ^18^ ^]^ This self‐organization is crucial for the successful development of functional HFs, which are essential for effective hair regeneration.^[^ ^19^, ^20^ ^]^ Despite these advancements, the field still faces challenges related to the scalability and reproducibility of BHG constructs. Addressing these issues is vital for translating laboratory success into clinical applications that can relieve individuals experiencing hair loss.

An appealing option is choosing water‐in‐water (W/W) emulsions, especially those incorporating poly(ethylene) glycol and dextran, as they are nontoxic and compatible with various biological applications.^[^ ^21^, ^22^ ^]^ Despite advancements in water‐droplet generation, using poly(ethylene) glycol and dextran to maintain long‐term dual‐layered cell encapsulation continues to be a significant challenge. Additionally, limited research has focused on the use of microfluidics to make the two aqueous phases in dual‐layered cell encapsulation systems more biocompatible and immiscible. These systems hold potential for use in applications requiring sustained cell encapsulation within dual layers, such as regenerating organoids or reconstructing organs in vivo.

Dual aqueous systems form through phase separation driven by repulsive interactions between the constituent aqueous phases.^[^ ^23^ ^]^ This separation occurs due to the thermodynamic incompatibility between a charged polysaccharide and a similarly charged protein.^[^ ^24^, ^25^ ^]^ Additionally, differences in molecular weight, such as those between a phase rich in macromolecules and another with smaller molecules, further promote phase separation.^[^ ^25^, ^26^ ^]^

**Figure 1:**
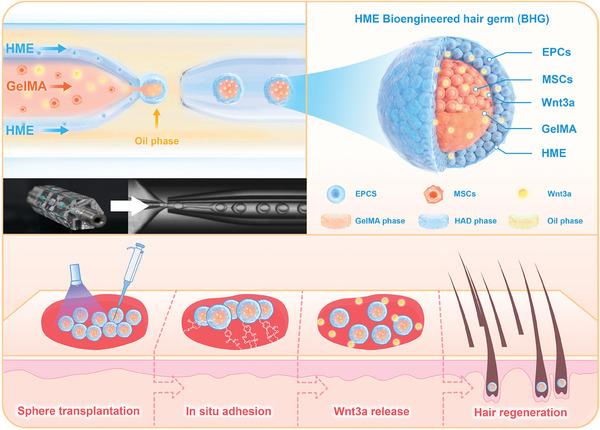
A schematic illustrating the preparation of bioengineered hair germs (BHGs) in large scale. Dihydroxyphenylalanine‐grafted hyaluronic acid (HME) and gelatin methacrylate (GelMA) were designed to encapsulate mesenchymal cells (MSCs) and epidermal cells (EPCs) via a co‐flowing microfluidic chip. Key features depicted include aqueous phase separation, wet adhesion properties, prolonged Wnt3a release, and enhanced hair follicle (HF) induction potential.

In our previous study,^[^ ^27^ ^]^ double‐T microfluidic chips have been used to generate dual‐layered cell microspheres, yet their application has been limited by strict flow rate control requirements, which often lead to issues such as improper encapsulation or multiple inner droplets being trapped. These challenges significantly restrict the reproducibility and scalability of the process. In this study, we address these limitations by utilizing a novel BHG coaxial‐flow microfluidic system, which comprising HME hydrogels and microfluidic‐generated microspheres encapsulating Wnt3a (**Figure** **1**). This design allows for simultaneous shearing of the three‐phase fluids, ensuring consistent encapsulation of a single inner droplet and improving the stability and efficiency of the microsphere‐generation process. The HME hydrogels provided a supportive and adhesive scaffold to achieve thermodynamic incompatibility with gelatin methacrylate (GelMA), whereas the microfluidic system enabled the production of uniform dual‐layered microspheres with controlled degradation and sustained Wnt3a release. The innovation enhances the scalability and practicality of the system for large‐scale hair regeneration applications. We hypothesized that this system may create a favorable microenvironment for HF regeneration by promoting MSC differentiation and activating key signaling pathways.

To test this hypothesis, we characterized the physicochemical properties of HME hydrogels, evaluated the morphology and degradation of microspheres, and assessed the sustained release of Wnt3a. Additionally, we conducted in vitro and in vivo experiments to analyze the impact of the system on HF regeneration, focusing on gene expression profiles, pathway activation, and functional outcomes.

## 2 Results and Discussion

### 2.1 HME Hydrogel Characterization

**Figure 2:**
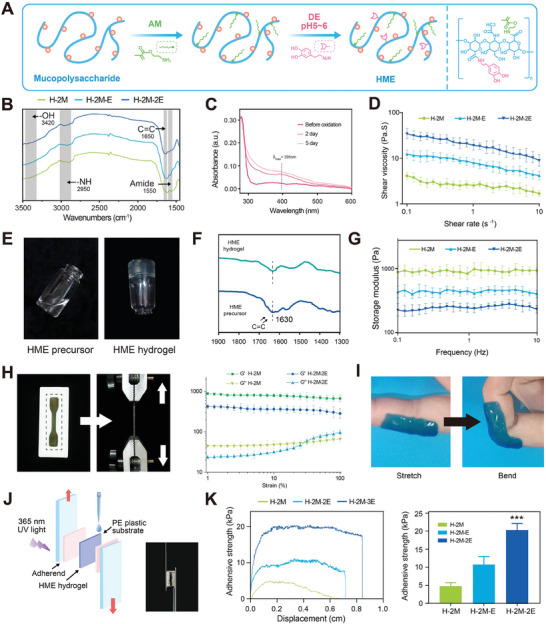
Characterization of mucopolysaccharide‐based hydrogels (HME). A) Synthesis of HME polymer. B) FTIR spectra of HME precursors. C) Ultraviolet–visible spectra of HME precursors after oxidation in air at 37 °C. Catechol peak: *λ*_max_(*ε*) = 275 nm. Quinone peak: *λ*_max_(*ε*) = 395 nm. D) Variation in shear viscosity with shear rate (0.1–10 s^−1^). *n* = 12. E) HME photo‐crosslinking and gelation. F) Comparison of FTIR spectra between photo‐crosslinked HME hydrogel and HME precursor. G) Frequency‐dependent rheological test of HME hydrogels with a frequency sweep from 0.1 to 10 Hz and a fixed strain amplitude of 1% at 37 °C, *n* = 12. H) Rheological tests with a strain‐dependent (1–100%) oscillatory measurement of HME hydrogels, the storage modulus (*G*′) and loss modulus (*G*″) were recorded. I) Adhesion state of HME hydrogels in different compression and relaxation skin folding states. J) Diagram of the lap shear test using porcine skin showing wet adhesion ability. K) The lap‐shear adhesive strength between porcine skin and HME hydrogels was quantitatively measured. *n* = 12. **p < 0.01 compared to all other groups.

Hyaluronic acid (HA), the primary component of the extracellular matrix (ECM), is widely distributed throughout the human body. The backbone of HME is composed of HA, an anionic, non‐sulfated glycosaminoglycan macromolecule consisting of repeating disaccharide units of d‐glucuronic acid and d‐*N*‐acetylglucosamine. The HME precursor was synthesized through attaching 2‐aminoethyl methacrylate (AM) and 3,4‐dihydroxyphenylalanine (DE) to HA polymer backbone (**Figure** **2**A), thereby endowing the material with wet adhesion and photocuring properties, respectively.^[^ ^27^, ^28^ ^]^ Cell migration assay (Figure S1, Supporting Information) and cell immigration‐related gene expression analysis (Figure S2, Supporting Information) results showed that 3.0 wt% HME is the optimal concentration for MSC migration. To optimize the cell immigration conditions, the HME concentration was set at 3.0 wt%. In the Fourier transform infrared (FTIR) analysis, absorption bands at 1650 and 1550 cm^−1^ correspond to the vinyl and amide groups on the mucopolysaccharide chains, respectively (Figure 2B). These results confirmed that AM and DE groups are successfully grafted, with varying mass ratios, onto HA macromolecules. Ultraviolet–visible (UV–vis) spectroscopy analysis revealed gradual HME oxidation of the catechol groups, with the lack of a quinone peak observed after 3 day oxidation (Figure 2C). Additionally, the HME solution viscosity demonstrated shear‐thinning behavior, decreasing as the shear rate increased in an exponential way (Figure 2D). These findings indicates that the HME precursor well‐suited for use in aqueous solutions.

Through brief ultraviolet exposure (4–5 s), we demonstrated the effective photo‐crosslinking of HME hydrogels using lithium phenyl‐2,4,6‐trimethylbenzoylphosphinate (LAP) as a photoinitiator (Figure 2E). In the photo‐crosslinked HME, the reduction of C═C bond content confirmed the formation of the AM network (Figure 2F). This reduction suggests that the crosslinking reaction between acrylate groups proceeded efficiently under UV exposure, resulting in a stable hydrogel network. Furthermore, the reduction in unsaturated bonds also indicates minimized residual monomer content, which can reduce potential cytotoxicity, further supporting the biocompatibility of the HME hydrogel for biomedical applications. Rheological analysis further showed that a lower DE concentration or higher AM concentration increased the storage modulus (*G*′) of HME hydrogels (Figure 2G). Additionally, the H‐2M‐2E hydrogel exhibited a loss modulus lower than its storage modulus (Figure 2H), indicating the retention of gel‐like behavior. These findings demonstrate that the AM network effectively maintained the structural integrity of the HME hydrogels across the tested strain and frequency ranges. This combination of structural integrity and shear‐thinning properties is particularly advantageous for applications such as injectable hydrogels or microfluidic cell encapsulation. This property allows the hydrogels to maintain a flowable state under high shear rates, such as during extrusion or microfluidic processing, which facilitates the construction of uniform microspheres. However, further optimization may be required to evaluate its performance under dynamic in vivo conditions. A microporous structure was observed in the HME hydrogel formulation through microscopic analysis (Figure S3, Supporting Information). This structural feature facilitates the removal of metabolic waste, supports signal exchange, and enhances nutrient transport. This structural feature resembles the pore architecture of polymer microspheres (such as polylactic acid),^[^ ^29^ ^]^ but the use of HA‐derived hydrogels may provide a more biocompatible alternative due to their natural ECM mimicry.

HME polymers exhibit strong tissue adhesion owing to the strong reactivity of catechol groups with nucleophiles on tissue surface proteins, including amines, thiols, and amide bonds.^[^ ^30^ ^]^ HME hydrogels, with a 8 s gelation time, adhered effectively to the skin, maintaining skin‐fold stability during joint movements (Figure 2I). To evaluate the adhesive strength between the skin and HME hydrogels, a lap‐shear test was conducted (Figure 2J). Higher DE concentrations significantly enhanced the adhesive strength compared to lower concentrations (*p* < 0.01) (Figure 2K). This improvement likely results from the increased density of catechol groups, which form robust covalent and noncovalent bonds with tissue nucleophiles, thereby reinforcing wet adhesion. Notably, H‐2M‐2E demonstrated an adhesive strength of ≈19.6 kPa (Figure 2K), significantly surpassing that of commercially available fibrin glue.^[^ ^31^ ^]^ Thus, H‐2M‐2E polymers were selected for preparing HME solutions for subsequent experiments. Cell Counting Kit‐8 assays and Live/Dead cell staining (Figure S4A–C, Supporting Information) showed good biocompatibility between cells and all HME samples. Hemolysis assays showed hemolysis ratios of <5%, indicating a minimal hemolytic effect of HME (Figure S4D, Supporting Information). Then GelMA was synthesized (Figure S5A, Supporting Information), and the methacrylation degree of GelMA was determined by proton nuclear magnetic resonance (^1^H‐NMR) spectrometry (Figure S5B, Supporting Information). The new signals from *δ* = 5.4 and 5.7 ppm that correspond to the acrylic protons of methacrylic functions from the methacrylic anhydride (MA) structure validate the synthesis of GelMA (Figure S5B, Supporting Information).^[^ ^32^, ^33^ ^]^ The methacrylation degree of GelMA was 67%. Figure S5C (Supporting Information) shows a Young's modulus value of *E* = 4.02 kPa for GelMA with 0.5% LAP. The equilibrium swelling ratio of the 7.5 wt% GelMA scaffold was near 165% (Figure S5D, Supporting Information). The rheological tests also showed the stability of the crosslinked hydrogels in the considered frequency range (Figure S5E, Supporting Information). The combination of ultrafast gelation, wet adhesion, shear‐thinning properties, and biocompatibility renders HME hydrogels suitable for applications in microfluidic cell encapsulation.

### 2.2 Characterization of Microfluidic Microspheres

**Figure 3:**
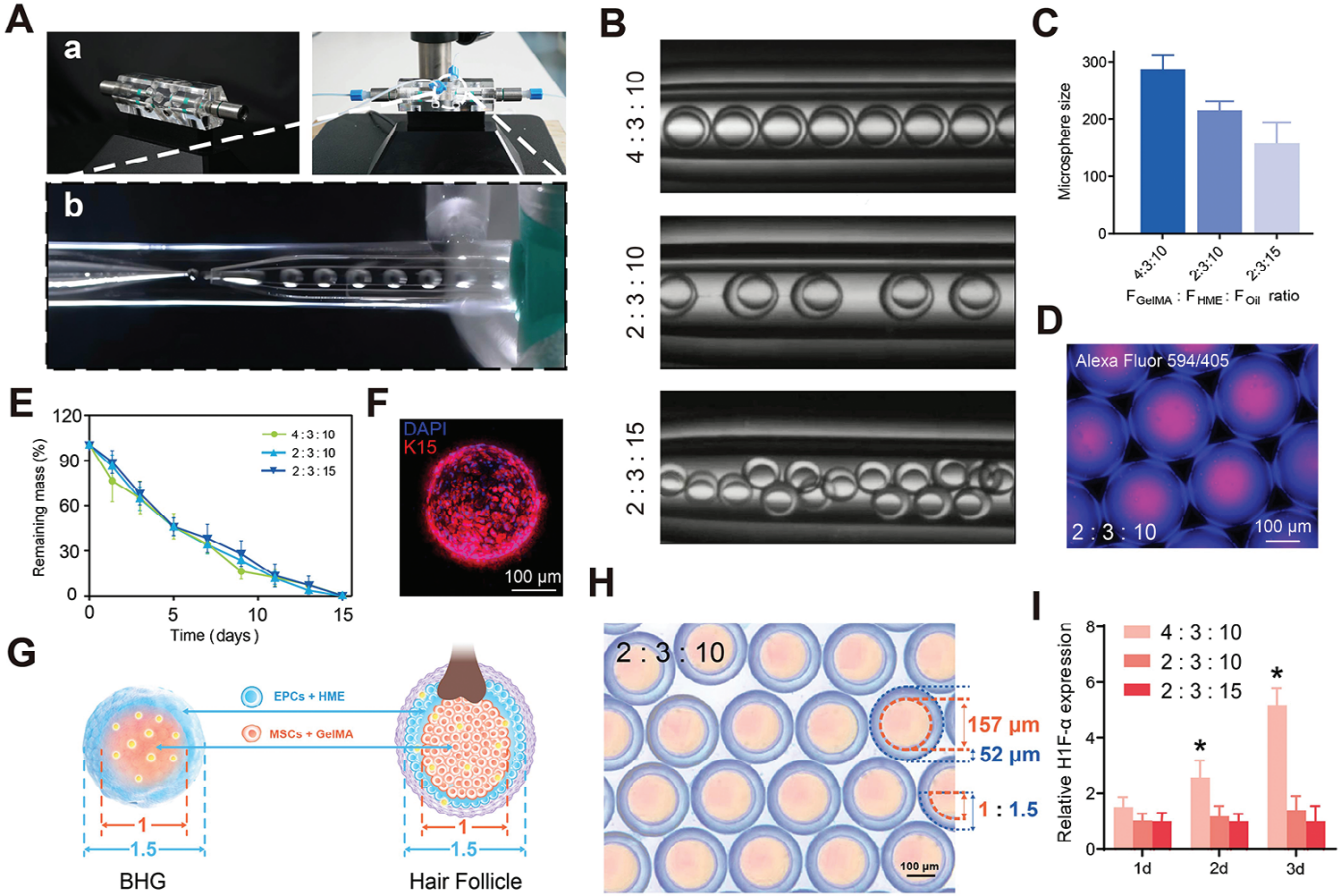
Microfluidic preparation of HME BHGs. A) The overview of co‐flowing BHG preparation systems. B) Photos of dual aqueous spheres formed at different flow rate ratios. C) Statistics of droplet size generated at different flow rate ratios. D) Microscopic image of Alexa Fluor 594/405‐dyed photo‐crosslinked double‐layered spheres. E) Degradation rate of HME spheres generated at different flow rate ratios (*n* = 16). F) Microscopic image of K15 and NucBlue‐labeled HME cell spheres after culturing for 24 h. G) Diagrams of biomimetic microfluidics simulating the proportional widths of hair follicle (HF) epithelium and dermis under physiological conditions. H) The inner‐to‐outer diameter ratio of the core–shell structure in microfluidic GelMA/HME. I) Relative expression of *HIF‐1α* by HME cell spheres generated at different flow rate ratios. *n* = 9. Scale bar: 100 µm.

A co‐flowing microfluidic BHG preparation system was developed (**Figure** **3**A‐a) for efficient and consistent generation of core–shell microfluidic droplets. This microfluidic chip included three inlet channels and one outlet, and its co‐flowing junctions were engineered to produce dual‐layered microdroplets (Figure 3A‐b). GelMA, known for its biocompatibility and biodegradability, facilitates cell adhesion,^[^ ^34^ ^]^ making it ideal for use in the inner phase. Given that the stiffness of biomaterials critically influences cell behavior and morphology, an HME precursor was utilized as the middle water phase. This choice aimed to replicate the mechanical characteristics of BHG tissue and maintain the sphere shell's adhesion in vivo.

Dual aqueous microfluidic droplets can be formed through interactions between charged high‐molecular‐weight polysaccharides and similarly charged proteins. To achieve this, a 7.5 wt% GelMA aqueous solution combined with 0.5 wt% LAP was selected for the internal phase, which also incorporated MSCs at a concentration of 1 × 10^7^ mL^−1^. GelMA was chosen for its ability to provide a biomimetic extracellular matrix environment that supports cell attachment and growth, making it an ideal candidate for encapsulating MSCs. Recognizing the significant role of material stiffness in modulating cellular morphology and behavior, we further developed a 3.0 wt% HME biomaterial for the middle phase. This phase was designed to not only enhance the mechanical stability of the droplet shell but also closely mimic the mechanical properties of HF tissue, thereby promoting effective cellular interaction and adhesion. EPCs were introduced into this phase at the same concentration (1 × 10^7^ mL^−1^), with the resin containing 0.1 wt% LAP to facilitate photoinitiated crosslinking. This design not only ensures optimal in vivo adhesion properties of the spherical shells but also closely replicates the mechanical attributes of HF tissue. The external phase consists of mineral oil supplemented with 2% w/v Span 80, which served to stabilize the dual aqueous droplets during formation. This formulation provides effective emulsification and prevents coalescence, ensuring consistent droplet generation.

Within this system, the inner GelMA and HME layers are broken down into bilayer droplets through shearing forces at the interface with the surrounding oil flow (Figure 3A‐b). This process relied on precise flow control achieved via three micropumps, while a high‐speed camera captured the droplet formation in real time, ensuring accurate monitoring of the process (Figure 3B). To adjust the droplet diameter, we varied the inner phase flow rate (*F*_GelMA_) between 1.0 and 2.0 mL h^−1^, maintained the middle phase flow rate (*F*_HME_) at 1.5 mL h^−1^, and adjusted the oil phase flow rate (*F*_oil_) from 5.0 to 7.5 mL h^−1^. These adjustments resulted in HME shell diameters ranging from 159.9 to 286.3 µm (Figure 3C). This level of control demonstrates the versatility of the platform in tailoring sphere size for specific applications, such as drug delivery or tissue engineering.

Using LAP as the photoinitiator, the GelMA/HME (HME) cell spheres were crosslinked with ultraviolet light for only 5 s to form stable structures (Figure 3D). The crosslinking efficiency underscores the rapid and scalable nature of this method, a critical advantage for high‐throughput production. The negatively charged macromolecular mucopolysaccharide‐based HME in the outer phase and the negatively charged protein GelMA in the inner phase generated increased electrostatic repulsion, effectively preventing the mixing of the internal and intermediate aqueous phases. This immiscibility enables the formation of stable HME microspheres, allowing efficient crosslinking in subsequent steps. Overall, this microfluidic platform offers significant advantages, including effective preparation of double‐layered spheres and precise control over sphere size.

To mimic in vitro degradation, the HME spheres were immersed in collagenase I (2.5 U mL^−1^) and hyaluronidase (5.0 U mL^−1^), resulting in complete degradation by day 15 (Figure 3E). This degradation timeline closely aligns with the typical requirements for HF cell morphogenesis and tissue reconstruction applications,^[^ ^7^, ^35^ ^]^ making it a relevant and practical model for studying tissue remodeling in vitro. The high cell density of K15^+^ EPCs within the microspheres allowed these cells to be densely packed throughout the structure. After 3 days of culture, the EPCs exhibited a rounded morphology, indicative of active cell proliferation and spatial interaction within the sphere (Figure 3F). This demonstrates the capacity of the HME‐based microspheres to support cellular behavior and maintain structural integrity during in vitro tissue development.

The hair germ size plays a pivotal role in HF morphogenesis, with sizes of >200 µm being required to ensure sufficient MSCs for effective cell–cell communication.^[^ ^36^ ^]^ The diameter ratio of the outer epithelial diameter to inner mesenchymal diameter is about 1.5:1. Additionally, in adult C57BL/6 mice, the hair bulb diameter generally does not exceed 280 µm (Figure 3G).^[^ ^37^ ^]^ By maintaining a flow rate ratio of 2:3:10 for the GelMA, HME, and oil phases, HME microdroplets with an average diameter of 221.2 µm were produced after rapid gelation. This configuration effectively replicates the cell density and scale of natural hair bulbs. Fluorescent images showed a distinct distribution of EPCs (core) and MSCs (shell) within the microspheres using a 2:3:10 flow rate ratio (Figure S6A, Supporting Information). By adjusting the flow rate ratio, we observed an increase in the microsphere diameter, with sizes exceeding 400 µm (Figure S6B, Supporting Information). After a 12 h incubation in complete culture medium, the size of the photo‐crosslinked cell spheres increased, reaching about 261.4 ± 19.5 µm diameter (Figure 3H). The HME shell exhibited an average thickness of 52.4 µm, yielding a shell‐to‐core diameter ratio close to 1.5:1 (Figure 3H; Figure S7, Supporting Information). This growth was primarily driven by mild swelling. The microfluidic platform demonstrated the consistent production of uniform microspheres both within individual batches and across different batches, with ≈320 cell spheres generated per minute. The spherical roundness and size were carefully regulated to ensure reproducibility. The HME cell spheres displayed aqueous phase separation, facilitating their use in microfluidic cell encapsulation.

Additionally, to evaluate the fluid exchange capability and the transport of oxygen and metabolites in the HME biomaterial scaffold, tissue hypoxia at the multicellular level was assessed by reverse transcription quantitative polymerase chain reaction (RT‐qPCR). After 2 days of culture, The expression of hypoxia‐inducible factor‐1 (*HIF‐1α*) significantly elevated in HME cell spheres prepared at a flow ratio of 4:3:10 after culturing for 2 days, compared to those fabricated at a flow ratio of 2:3:10 (Figure 3I). This increase may be attributed to an elevated cell density or a larger microsphere diameter, both of which could influence cellular metabolism and oxygen consumption. These findings suggest that a ratio flow of 2:3:10 is optimal for generating BHGs, balancing cell viability and metabolic activity.

To explore the cell viability within the microspheres, Live/Dead staining showed good cell survival after in vitro culturing for 9 days (Figure S8A,B, Supporting Information). Immunofluorescence staining confirmed the presence of cell adhesion complex *Ctnnb1* in HME cell spheres (Figure S8C, Supporting Information), suggesting proper cell adhesion and interaction within the microspheres. RT‐qPCR results indicated a significantly lower expression of HIF‐α in the HME microspheres compared to the hanging drop (HD) microspheres, further supporting the favorable microenvironment provided by the HME microspheres for cell growth (Figure S8D, Supporting Information).

Taken together, these results suggest that mucopolysaccharide‐based HME shells with GelMA cores exhibit thermodynamic incompatibility, leading to aqueous phase separation in HME cell spheres. This property is critical for the stability and controlled release of oxygen and nutrients. To conclude, the dual aqueous cell spheres generated by this microfluidic system proved to be both efficient and straightforward, potentially providing a biocompatible microenvironment conducive to MSC and EPC activity for promoting hair regeneration.

### 2.3 Sustained Wnt3a Release of Microfluidic BHG Spheres

The HF mesenchyme can physically interact with the mantle epithelium in its physiological context, thus becoming a key signaling hub for HF development.^[^ ^38^ ^]^ Among the key mediators, Wnt3a is thought to play a crucial role in maintaining the hair‐inducing properties of the mesenchyma.^[^ ^39^ ^]^ The Wnt3a signaling pathway is integral in regulating cell fate decisions and promoting epithelial–mesenchymal cross‐talk, crucial for initiating and maintaining hair follicle formation.

**Figure 4:**
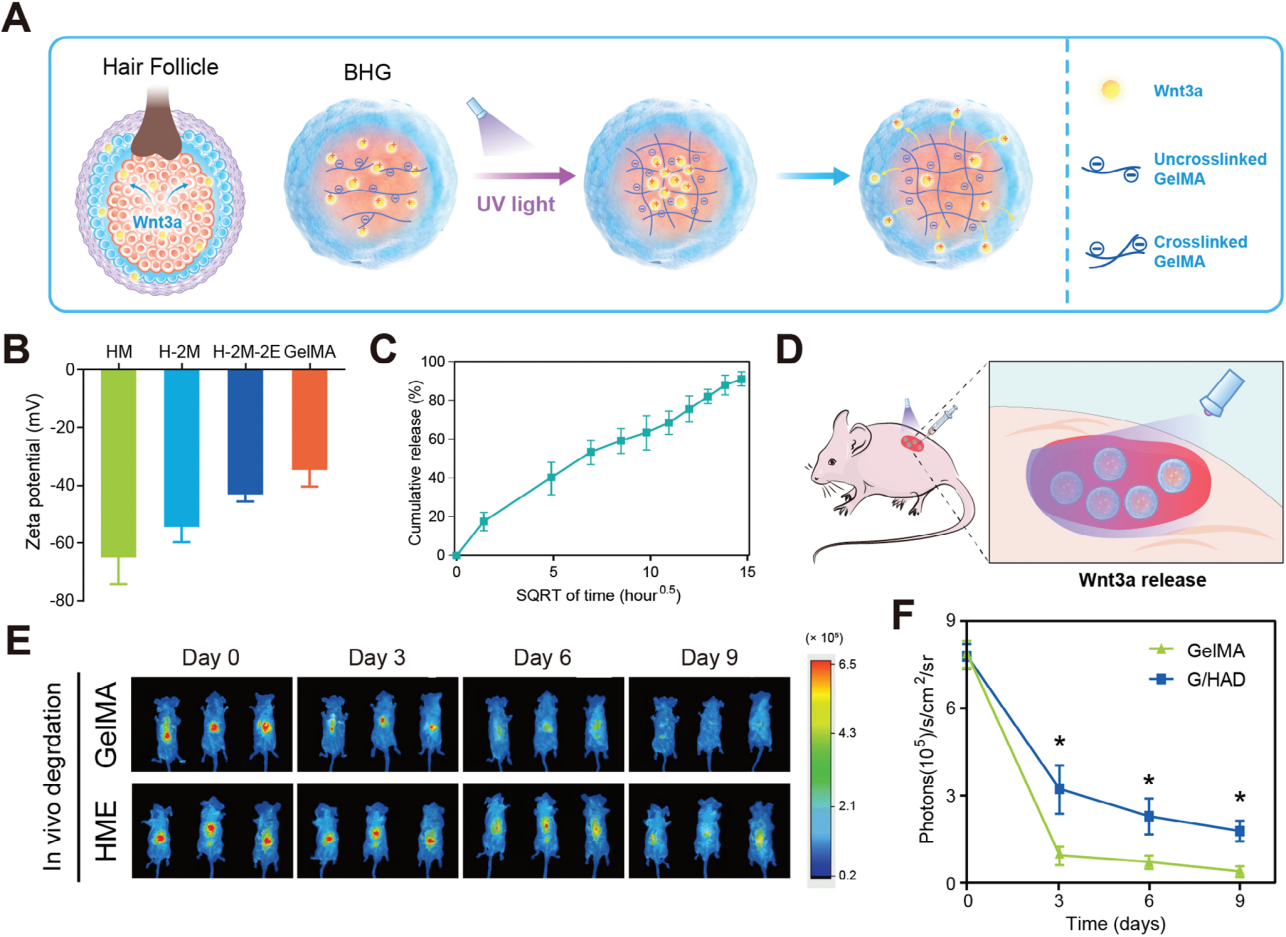
Sustained in vivo and in vitro Wnt3a release mechanism. A) The sustained release of Wnt3a emulates hair germ dermal–epithelial cell–cell communication. B) Zeta potential of HM, HME, and GelMA precursor at pH 7.4. *n* = 16. C) Cumulative Wnt3a release with square root of time from HME microspheres (*n* = 16). D) Schematic of Wnt3a encapsulated microfluidic sphere transplantation. E) Wnt3a sustained release by microfluidic GelMA and HME spheres. F) Quantitative analysis of Wnt3a fluorescence signal intensity. Data are expressed as means ± SD. *n* = 12. *p < 0.01.

Unlike traditional biomaterials such as silk polypeptides, collagen, Matrigel, and fibroin, both GelMA and HME are capable of phase separation in aqueous solutions, making them suitable for use in microfluidic systems. Nevertheless, the establishment of a successful HF regeneration culture system still lacks essential growth factors. To address this issue and better emulate the interaction between mesenchyme and epithelium,^[^ ^7^ ^]^ a GelMA core‐based sustained‐release model for Wnt3a was developed (**Figure** **4**A). The isoelectric point (pI) of the GelMA scaffold is pH 5,^[^ ^31^ ^]^ with a zeta potential of −35.3 ± 7.59 mV (Figure 4B), rendering it negatively charged at physiological pH. In contrast, Wnt3a has a pI of pH 9.6, making it positively charged. Based on these properties, we hypothesized that ionic interactions between the crosslinked GelMA network and Wnt3a would contribute to the sustained release of Wnt3a. To evaluate release efficiency, HME spheres encapsulated with Wnt3a at a concentration of 200 ng mL^−1^ were prepared. We assessed the encapsulation and release efficiency of Wnt3a by enzyme‐linked immunosorbent assay (ELISA). The encapsulation efficiency of Wnt3a was ≈90%. Regarding release efficiency, the limited binding affinity of Wnt3a to the GelMA scaffold surface may account for the immediate release of ≈20% of the initially loaded Wnt3a. By day 7, nearly 90% of Wnt3a had been released, with the in vitro release lasting ≈9 days (Figure 4C). A plot of Wnt3a release versus square root of time demonstrated a linear relationship (Figure 4C), suggesting a diffusion‐controlled mechanism in the release process. It is crucial for MSC‐mediated hair growth that these findings underscore the role of ionic interactions involving Wnt3a and the GelMA backbone.

To determine whether HME spheres are capable of the sustained release of Wnt3a in vivo, both GelMA spheres and dual layered GelMA/HME (HME) spheres loaded with dye‐labeled Wnt3a were fabricated, transplanted into the dorsal dermis of nude mice (Figure 4D), and imaged by In Vivo Imaging System (IVIS) (Figure 4E). Intense fluorescence signals were detected on the transplant site with Wnt3a‐loaded spheres on day 0. However, Wnt3a signals showed significant diffusion on day 3 and diminished to nearly undetectable levels by day 6 in the GelMA sphere group. This could be attributed to the fragmentation, translocation, and diffusion of a GelMA sphere. This indicates that the GelMA sphere is unsuitable for in vivo transplantation because of its low modulus and lack of wet tissue adhesion properties (Figure 4E). These findings suggest that GelMA spheres alone are unsuitable for in vivo applications requiring prolonged retention.

In contrast, HME spheres demonstrated a more concentrated fluorescence signal at the transplantation site, persisting until day 9 before showing significant attenuation without observable diffusion. This extended retention period, longer than that of most microfluidic spheres, highlights the potential of HME spheres for sustained Wnt3a release during the early stages of HF regeneration.^[^ ^39^ ^]^ Quantitative analysis of fluorescence intensity showed that Wnt3a signals exhibited greater longevity in HME microspheres compared to GelMA microspheres (*p* < 0.01) (Figure 4F). This improvement can be attributed to the presence of DE groups in the HME hydrogel, which enhances the adhesion and stability of the microspheres within the transplantation site. These results underscore the advantage of HME microspheres in maintaining Wnt3a stability and prolonging its localized release, making them a promising delivery vehicle for applications in HF regeneration.

### 2.4 Profiling of Gene Expression of BHG Microspheres

**Figure 5:**
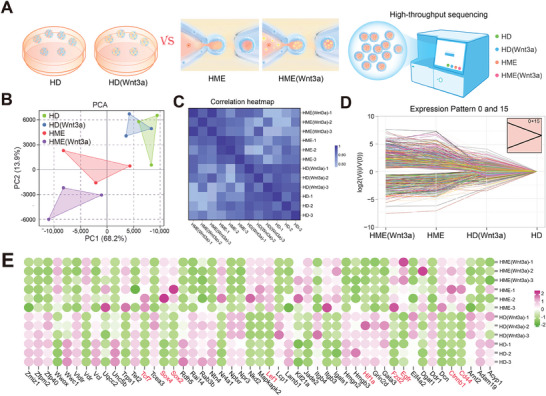
Transcriptional analysis demonstrating the influence of the HME microenvironment and Wnt3a release. A) RNA‐seq of traditional HD cell spheres and microfluidic HME cell spheres with or without Wnt3a encapsulation. B) PCA analysis of overall gene expression per sample. C) Pearson's correlation computation of transcriptome data in all samples. D) Patterns of gene expression indicating progressive upregulation or downregulation of genes across groups. E) Heatmap displaying the top 60 progressively regulated genes with the highest enrichment.

To investigate the impact of Wnt3a release on cellular behavior within the HME scaffold, we used the HME system and the previously described HD method,^[^ ^40^ ^]^ dividing them into groups with or without Wnt3a encapsulation to extract messenger RNA (mRNA) for evaluating the impact of Wnt3a release with HME scaffold. Gene expression profiles were assessed in the HME (Wnt3a), HME, HD (Wnt3a), and HD groups by high‐throughput sequencing (**Figure** **5**A). Principal component analysis (PCA) was performed to assess intergroup and intragroup variations. The first two principal components accounted for 86.4% of the total variation in gene expression (PC1:68.2%; PC2:13.9%) (Figure 5B). It is worth noting that biological replicates demonstrated a high degree of correlation, validating the technical consistency of the profiling data. PCA showed distinct separation between groups, highlighting significant differences in gene expression. The high reproducibility of parallel samples was further confirmed by Pearson correlation coefficient analysis, which also showed the most significant gene expression pattern differences between the HD and HME(Wnt3a) groups (Figure 5C). The above results showed that the different culture conditions of HME and HD microspheres led to significant changes in their gene expression patterns.

Thus, we focused on gene expression patterns among groups. Using short time‐series expression miner (STEM) analysis, we identified genes that exhibited similar expression patterns. Significant enrichment (*p* < 0.05) was observed in patterns 15 and 0, which included 1252 progressively downregulated genes and 397 progressively upregulated genes across the HME (Wnt3a), HME, HD (Wnt3a), and HD groups, respectively (Figure 5D). These enriched genes, along with Wnt3a delivery, may play pivotal roles in the biomimetic HME microenvironment. Remarkably, the top‐50 enriched genes in the heat map revealed significant upregulation of the HF regeneration‐related genes *Ctnnb1*, *Lef1*, and *Tcf7*, as well as hyaluronic acid receptor *Cd* ^[^ ^41^ ^]^ and epidermal growth factor receptor) (*Egfr*). These genes are known to play essential roles in promoting cell–cell communication, proliferation, and differentiation during HF regeneration.^[^ ^43^, ^44^ ^]^ Interestingly, the hypoxia‐related gene *Hif‐1α* was significantly downregulated in the HME (Wnt3a) group compared to other groups (Figure 5E). This finding suggests that the enhanced oxygen and nutrient exchange capabilities of the HME scaffold, coupled with the controlled release of Wnt3a, may alleviate cellular hypoxia, fostering a more conducive environment for HF regeneration.

### 2.5 Functional Analysis and Validation of Cell Microsphere

**Figure 6:**
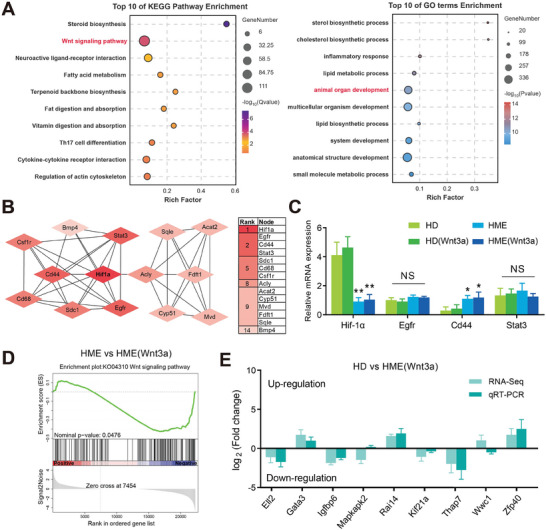
Functional analysis demonstrating the influence of the HME microenvironment. A) Kyoto Encyclopedia of Genes and Genomes (KEGG) pathway and Gene Ontology (GO) term analysis of genes in expression patterns 0 and 15. B) Cytohubba identified the hub genes. C) RT‐qPCR analysis of hub genes *Hif‐1α*, *Egfr*, *Cd44*, and *Stat3* (*n* = 12). *p < 0.05 and **p < 0.01 compared to HD group. D) GSEA enrichment analysis in HGM versus HGM (Wnt3a). E) RT‐qPCR verification of gene expression of randomly selected enriched genes (*n* = 12).

Functional analysis was conducted to investigate enriched genes identified in expression patterns 0 and 15. These genes were significantly enriched in Kyoto Encyclopedia of Genes and Genomes (KEGG) pathways associated with HF regeneration, such as the “Wnt signaling pathway,” which is a well‐known regulator of HF regeneration. Additionally, Gene Ontology (GO) term analysis revealed a strong association with “animal organ development,” indicating that the biomimetic microenvironment supports not only HF‐specific processes but also broader developmental pathways that may enhance the scaffold's regenerative potential (**Figure** **6**A). These findings suggest notable alterations in HF regeneration potential within the HME (Wnt3a) scaffold.

To explore interactions between genes with expression patterns 15 and 0, we constructed a protein–protein interaction (PPI) network using Molecular Complex Detection (MCODE). The analysis identified a high‐scoring module with 14 nodes and 41 edges, pinpointing *Hif‐1α* as a central hub gene using CytoHubba (Figure 6B). This finding underscores the pivotal role of *Hif‐1α* in the regulatory network associated with HF regeneration. Moreover, RT‐qPCR analysis confirmed that the expression levels of these hub genes were consistent with the sequencing results after 3 days of culture (Figure 6C). Furthermore, compared with traditional HD cell spheres, HME cell spheres exhibited significant upregulation of the hyaluronic acid receptor *Cd44*, indicating that HME scaffolds enhance and maintain the cellular microenvironment. Additionally, *Hif‐1α* expression was notably downregulated, suggesting that the HME scaffold effectively alleviated cellular hypoxic conditions. While there were no significant differences in the expression levels of *Egfr* and *Stat3* between groups. Enhancement of the cellular hypoxic microenvironment and cytokine metabolism plays crucial roles in HF regeneration, ultimately contributing to improved hair regeneration efficiency. We performed gene set enrichment analysis (GSEA) on the profiling data from the HME and HME (Wnt3a) groups to identify specific pathways influenced by sustained Wnt3a release. The KEGG Orthology (KO) term “Wnt signaling pathway” was significantly upregulated in the HME (Wnt3a) group (Figure 6D). To validate the transcriptome profiles, we randomly selected nine enriched genes and conducted RT‐qPCR analysis. The results showed similar trends with RNA‐seq profiling data (Figure 6E), confirming the reliability and consistency of our RNA‐seq results. These findings suggest that the HME (Wnt3a) scaffold creates a more favorable environment for HF development. These results showed HME scaffold a favorable environment for HF development and enhance regeneration outcomes. In summary, the HME (Wnt3a) scaffold fosters a more favorable microenvironment for HF development by mitigating hypoxia, supporting cytokine metabolism, and activating key pathways like Wnt signaling. These results highlight its potential to enhance hair regeneration efficiency by targeting both molecular and cellular mechanisms. Other organoid technologies, such as fibrin‐based scaffolds,^[^ ^42^ ^]^ tumor organoid,^[^ ^43^ ^]^ and 3D bioprinting platforms,^[^ ^44^ ^]^ have demonstrated the ability to preserve the structural and functional properties of original tissues, highlighting their utility as models for studying developmental mechanisms and therapeutic interventions. Translating this principle to hair follicle organoids could offer a valuable platform for investigating folliculogenesis and related disorders, such as alopecia or scarring alopecia. These models could also serve as testing grounds for novel drugs, offering a personalized approach to hair regeneration.

### 2.6 HME Microsphere Transplantation and In Vivo Regeneration

**Figure 7:**
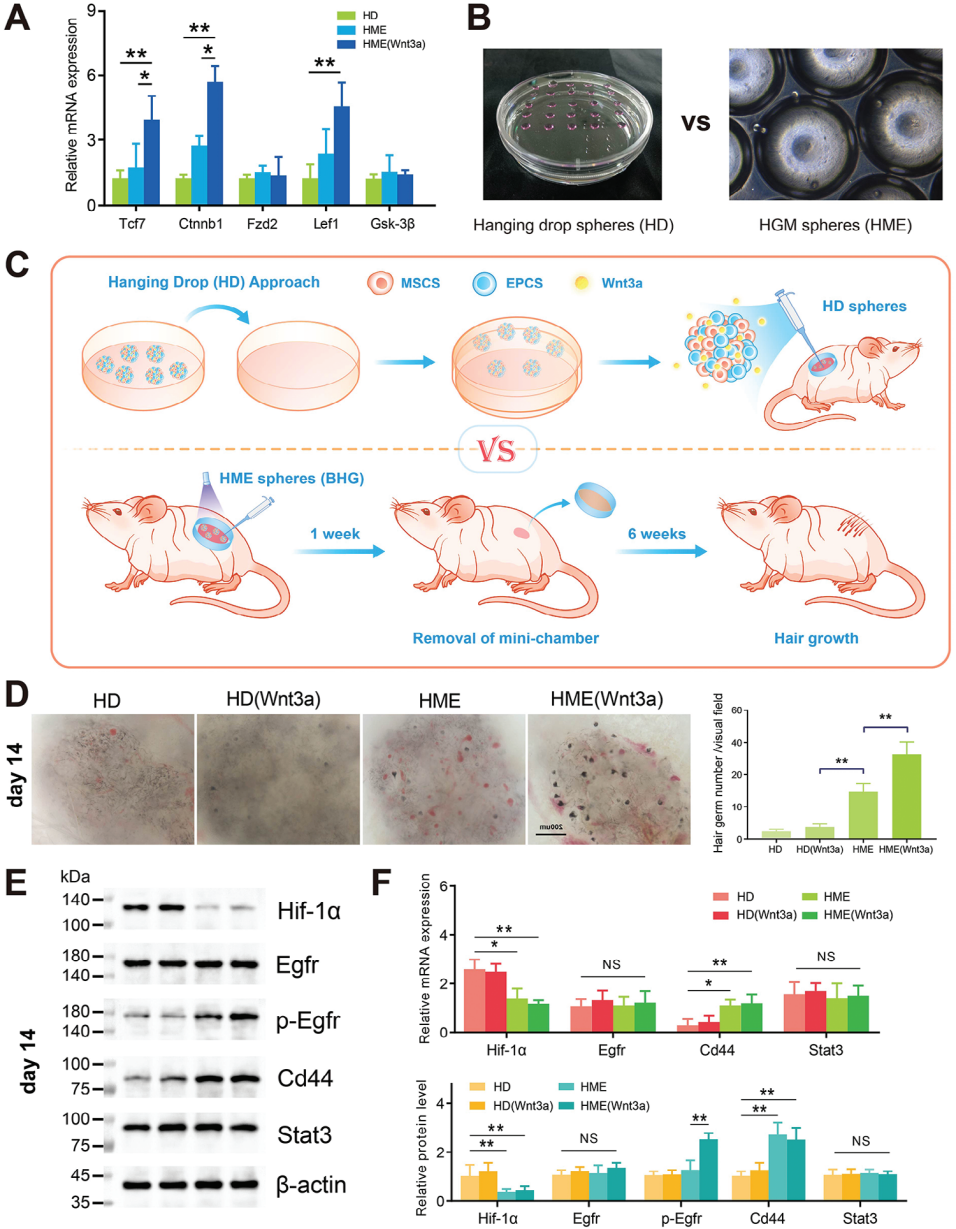
In vivo BHG transplantation and regeneration. A) RT‐qPCR analysis of known follicle‐regulatory genes in cultured cell spheres on day 3 (*n* = 12). B) Pre‐transplantation photographs of hanging drop (HD, as negative control) and double‐layered microfluidic cell spheres. C) Diagram illustrating the transplantation of BHG spheres, employing the chamber to promote in situ longitudinal hair induction. HD cell spheres were also transplanted as a negative control. D) Macroscopic observations of the regenerated primary hair germs on day 14. Scale bar: 200 µm. E) Western blot reveals changes in protein expression of hub genes on day 14. Full‐length blots/gels are presented in Figure S10 (Supporting Information). F) RT‐qPCR and western blot analysis of hub gene expression on day 14. Relative mRNA and protein expression levels were normalized to β‐actin. *p < 0.05, **p < 0.01 (*n* = 9 individual experiments).

After 5 days of culture, the cell microspheres were analyzed using RT‐qPCR to invalidate how the model influences hair regeneration‐related genes (**Figure** **7**A). The expression levels of *Tcf7*, *Ctnnb1*, and *Lef1* were significantly higher in this group than in the other, underscoring the activation of the Wnt signaling pathway. This pathway is critical for early HF morphogenesis, as it facilitates the rapid transition of hair germ structures into anagen phase hair follicles.^[^ ^45^ ^]^ These findings highlight the synergistic role of Wnt3a within HME microspheres in boosting hair‐inductive potential.

Lichti's chamber assay was performed to assess the in vivo regenerative potential of HF.^[^ ^45^ ^]^ HD and HME microspheres were transplanted into minichambers implanted under dorsal skin of BALB/c nude mice (Figure 7B,C). Analysis of the number of regenerated hair germ cells after 14 days revealed new hair germ formation, with the largest number observed in the HME (Wnt3a) group (Figure 7D). On day 14, 33.6 ± 9.5, 14.4 ± 3.6, 4.1 ± 1.2, and 2.7 ± 0.6 regenerated hair germs per visual filed were observed in the HME (Wnt3a), HME, HD (Wnt3a), and HD groups, respectively (Figure 7D). Hair germs stimulated by HD spheres were more dispersed and disordered, which may have been caused by the translocation of HD cell spheres with movement of the nude mice. In addition, owing to the superior mechanical properties and wet‐tissue adhesion ability of HME shells, HME cell spheres could better resist skin movement and fluid infiltration, allowing for stable dermal implantation without fragmentation or displacement.

RT‐qPCR and western blot analysis were conducted to examine hub gene expression in vivo. There was a significant increase in Hif‐1α expression in hanging drop spheres compared with the HME spheres on day 14 (Figure 7E,F). However, the expression of the Cd44 in the skin at the graft site of nude mice was significantly increased in the HME group, while western blot results showed that the phosphorylation levels of Egfr were significantly increased in the HME (Wnt3a) group (Figure 7E,F), indicating the promoting effect of HME scaffold and sustained Wnt3a release on HF regeneration in vivo. By improving the local microenvironment and promoting the activation of key signaling pathways, the HME system demonstrates a clear advantage over traditional HD spheres for HF regeneration applications.

**Figure 8:**
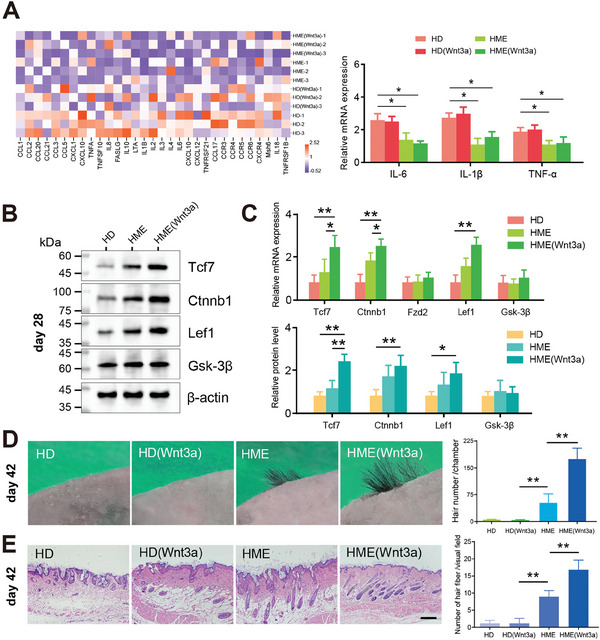
In vivo investigation of BHGs for the hair regeneration ability. A) PCR array and RT‐qPCR analysis showing expression of inflammation‐related genes in the HME group compared to the HD group on day 9 post‐transplantation. B) Western blot reveals changes in protein expression of HF induction‐related genes on day 28. Full‐length blots/gels are presented in Figure S10 (Supporting Information). C) RT‐qPCR and western blot analysis of HF induction‐related gene expression on day 28. Relative mRNA and protein expression levels were normalized to β‐actin. D) Photograph of regenerated hair from different groups. Bar graph of the number of regenerated HFs. E) H&E staining of longitudinal sections of HFs at the transplant site 6 weeks after transplantation. Scale bar: 100 µm. *p < 0.05, **p < 0.01 (*n* = 9 individual experiments). H&E, Hematoxylin and Eosin.

The PCR array analysis revealed that the expression of inflammation‐related genes was significantly lower in the HME group compared to the HD group (**Figure** **8**A). Consistent with this finding, results from RT‐qPCR (Figure 8B) and western blot assays (Figure S9A,B, Supporting Information) further confirmed that the expression levels of key inflammatory markers, including interleukin (IL)‐6, IL‐1β, and tumor necrosis factor‐alpha (TNF‐α), were significantly reduced in the HME group when compared to the HD group. GSEA and KEGG analyses also showed TNF signaling pathway significantly enriched involved in the response to HME treatment (Figure S9C,D, Supporting Information), further supporting the reduced inflammatory response observed in the HME group. RT‐qPCR and western blot analysis showed a significant increase in Wnt signaling pathway related gene Tcf7, *Ctnnb1*, and Lef1 activity in HME (Wnt3a) group on day 28 (Figure 8B,C). These findings suggest a potential mechanism for hair regeneration in vivo induced by HME (Wnt3a) microspheres.

About 6 weeks after transplantation, we observed that many normal functional HFs with associated black shafts grew in the direction of physical hair growth (Figure 8D). 171.3 ± 33.5, 56.2 ± 19.8, 1.7 ± 0.7, and 2.2 ± 0.8 regenerated HFs were observed in the HME (Wnt3a), HME, HD (Wnt3a), and HD groups, respectively (Figure 8D). Hematoxylin and Eosin (H&E) staining of longitudinal‐sectional tissue slices at the transplantation site 6 weeks post‐transplantation showed the highest HF‐stimulating efficacy for HME (Wnt3a) microspheres and the lowest for HD microspheres (Figure 8E). The outcome demonstrated that the released Wnt3a signals enhanced the HF induction efficacy of BHGs. This may be due to Wnt3a promoting the downstream Wnt signaling expression in BHGs, whereas the traditional HD microspheres lack biological scaffolds, preventing sustained release of Wnt3a. These results indicate that the microenvironment of HME (Wnt3a) spheres can improve hair germ regeneration.

The designed HME (Wnt3a) scaffold facilitated the encapsulation of EPCs and MSCs, leveraging the biomimetic structure to reduce cell consumption while preserving epithelial–mesenchymal interactions and ensuring adequate cell density.^[^ ^45^ ^]^ In fact, it requires a large quantity of epithelial cell suspension combined with MSC spheres in traditional hair regeneration models (Table S1, Supporting Information). However, the BHG spheres we used show significantly decreased cell consumption, with the cell usage being 1/13th of that in traditional minichamber assays.^[^ ^45^ ^]^ HME shells, with their high modulus and excellent tissue adhesion properties, ensured stable attachment of BHG spheres to dorsal skin of mice. This prevented displacement or breakdown within skin and maintained a stable HF induction effect. Adversely, the adhesion of traditional HD microspheres without biological scaffolds is unstable and fails to sustain the release of Wnt3a. These results emphasized the combined effect of the HME scaffold and Wnt3a, demonstrating the superior efficacy of microfluidic HME microspheres in promoting hair regeneration in vivo. The synergistic effect of Wnt3a with BHGs was confirmed by these results, which also demonstrated the high efficacy of BHG spheres in in vivo hair regeneration assays. Focused on improving hair regenerative medicine, this microfluidic approach is especially relevant for treating severe hair loss conditions, such as androgenetic alopecia.

Despite its promising results, this study had certain limitations. The in vivo experiments were performed in a controlled environment, and further investigation is needed to evaluate the long‐term efficacy and safety of the BHG system in various clinical settings. The incorporation of other growth factors or signaling molecules may further improve HF regeneration. Future optimizations could also include incorporating responsive materials that adapt dynamically to environmental stimuli. Further refinement of the W/W emulsion system may also enhance its ability to support long‐term cell function. Future studies should also focus on optimizing delivery systems and exploring their potential applications in other areas of regenerative medicine.

## 3 Conclusion

This study presents the successful development of a BHG using mucopolysaccharide thermodynamic incompatibility, which offers a novel approach for enhancing HF regeneration. HME hydrogels exhibit excellent physicochemical properties including rapid gelation, high wet adhesion, and biocompatibility. Using a co‐flow microfluidic system, dual‐layered microspheres with HME shells and GelMA cores were fabricated to provide a controlled microenvironment for sustained Wnt3a release.

The sustained release of Wnt3a effectively promoted HF morphogenesis and regeneration by activating the Wnt signaling pathway and regulating the expression of key genes such as *Ctnnb1*, *Lef1*, and *Cd44*, while downregulating hypoxia‐related pathways. Functional analyses confirmed the enrichment of HF regeneration pathways, further validating the regenerative potential of HME (Wnt3a) microspheres.

Overall, this study demonstrated that the BHG system can provide a biomimetic, sustained‐release microenvironment conducive to HF regeneration. This platform offers significant potential for clinical applications in hair loss treatment and transplantation, paving the way for advanced regenerative therapies.

## 4 Experimental Section

### Ethical Statement

All animal experiments were performed in accordance with the regulations set forth by the Institutional Animal Care and Utilization Committee of Southern Medical University. Ethical approval for the procedures was obtained from the Institutional Animal Care Committee (K2019032). Male BALB/c nude mice (4 weeks old, weighing 25–30 g) and female neonatal C57BL/6J mice (25–30 g) were procured from the Experimental Animal Centre at the Southern Medical University. The mice were maintained at 25 °C with controlled humidity and in groups of up to five under specific pathogen‐free conditions with a 12 h light/dark cycle, starting at 07:00 and ending at 19:00 for the light phase. The study also received approval from the Human Research Ethics Committee of Nanfang Hospital (NFEC‐2021‐218). All the participants provided written informed consent for participation in the study. All procedures adhered to the Declaration of Helsinki.

### Materials

Sodium hyaluronate used in this study was obtained from Freda Biochem (CAS: 9067‐32‐7, Freda, ChinaShandong, China). AM hydrochloride (#516155), ethyl(dimethylaminopropyl) carbodiimide (EDC) (#E7750), *N*‐hydroxysuccinimide (NHS) (#130672), and dopamine hydrochloride (#H8502) were purchased from Sigma–Aldrich (St. Louis, MO, USA). Fetal bovine serum (FBS) (#10270‐106), Alexa Fluor 594/405 Microscale Protein Labelling Kit (#A30008/A30007), and Live/Dead reagent (#L3224/C3099) were purchased from Invitrogen (Carlsbad, CA, USA). Recombinant Wnt3a (#5036‐WN‐010/CF) was purchased from R&D Systems, USA. Detailed reagents and materials list is shown in Table S2 (Supporting Information).

### MSC and EPC Isolation

Skin cells were harvested from newborn C57BL/6J mice as described.^[^ ^27^ ^]^ To isolate EPCs and MSCs, the skin of newborn C57BL/6J mice was first treated with 0.2% w/v dispase (# D4693, Sigma–Aldrich, USA) for 12 h at 4 °C, then rinsed three times with phosphate‐buffered saline (PBS) (#P3813, Sigma–Aldrich, USA). The epidermis was separated from the dermis using forceps. The epidermal layer was minced for 15 min and then incubated with 0.3% w/v trypsin (#T4799, Sigma–Aldrich, USA) for 6 min. The dermal layer was minced and subjected to a 30 min incubation with 0.3% (w/v) collagenase (#C0130, Sigma‐Aldrich, USA). The resulting cell suspensions were filtered through strainers, followed by centrifugation at 1000 × *g* for 5 min at 4 °C. The collected cells were resuspended for subsequent use.

### Hydrogel Preparation and Characterization

For hydrogel preparation, the HME polymer was to be synthesized; 1 g of HA was dissolved in 100 mL of deionizer water at a concentration of 1% w/v. NHS (4 mmol), EDC (4 mmol), and AM were sequentially added to the HA solution and stirred for 30–40 min. Dopamine hydrochloride (#H8502, Sigma–Aldrich, USA) was then added and the solution was allowed to react for 24 h. The entire process was carried out under a nitrogen atmosphere and kept in the dark at 25 °C to avoid oxidation. The pH was adjusted to 5–6 using 0.1 n sodium hydroxide (#S8045, Sigma–Aldrich, USA) and hydrochloric acid (#H1758, Sigma–Aldrich, USA). Unreacted monomers were removed by dialysis [Molecular Weight Cut‐Off (MWCO) = 8000–12 000 Da)] under acidic conditions for 72 h, followed by lyophilization. The resulting lyophilized polymer foam was stored in a sealed container until further use. The mass ratios of HA:AM:DE were adjusted to 1:2:0, 1:2:1, and 1:2:2, corresponding to polymers H‐2 M, H‐2M‐E, and H‐2M‐2E, respectively.

For GelMA synthesis, type A porcine skin gelatin (#G1890, Sigma–Aldrich, USA) was dissolved in PBS (pH 7.4) at 50 °C under continuous stirring to form a 10% (w/v) gelatin solution. Methacrylic anhydride (#760‐93‐0, Sigma–Aldrich, USA) was added drop wise to the gelatin solution at a volume ratio of 0.5 mL MA per 10 mL gelatin solution, maintaining the reaction temperature at 50 °C. The reaction was carried out for 3 h. The resultant solution was dialyzed against deionized water at 40 °C for 7 days. The purified GelMA was lyophilized for 5 days. The degree of methacrylation of GelMA was determined using ^1^H‐NMR spectroscopy. To prepare crosslinked hydrogels, GelMA was dissolved in PBS at 37 °C to form a 7.5% (w/v) solution. Lithium phenyl‐2,4,6‐trimethylbenzoylphosphinate (Sigma–Aldrich, USA) was added as a photoinitiator at a concentration of 0.1% or 0.5% (w/v). The solution was exposed to UV light (365 nm, 10 mW cm^−^^2^) for 15 s to achieve crosslinking. The swelling ratio of GelMA hydrogels was evaluated by immersing photo‐crosslinked GelMA samples in PBS (pH 7.4) at 37 °C. The swollen samples were blotted gently to remove excess liquid and weighed.

For the HME polymer and hydrogel characterization, the chemical structures of the HME polymers were examined using FTIR. FTIR spectra were recorded using a NICOLET 6700 instrument (Thermo Scientific, Rockford, IL, USA) within the 1500–3500 cm^−1^ range. For UV–vis spectroscopy, a 0.5 wt% HME hydrogel precursor solution was prepared, and UV–vis spectra were collected using a spectrophotometre (Evolution 60S UV–Vis Spectrophotometer/Evolution 200 UV‐Vis Spectrophotometer, Thermo Electron Corporation, USA) over the wavelength range of 200–650 nm. To measure the zeta potential, both HA and HME precursor solutions were dispersed in PBS (pH 7.4) for 10 min, filtered to remove larger charged particles, and then analyzed using dynamic light scattering (Zetasizer Nano ZS90, Malvern Instruments, UK) at 25 °C.

To start HME gelation, a 3.0 wt% precursor solution was prepared and mixed with 0.1 wt% LAP (#SE‐3DP‐0105, StemEasy, China), which acted as a photoinitiator. The hydrogel was formed by exposing the mixture to UV light at 365 nm for 4–5 s, triggering photo‐crosslinking. The resulting solution was then placed in cylindrical moulds (5 mm high and 20 mm in diameter) to create photo‐crosslinked HME hydrogels, which were subsequently utilized for rheological analysis. Rheological properties were measured using a Haake Mars 40 rheometer (Thermo Fisher Scientific, Waltham, MA, USA) equipped with a 20 mm diameter parallel‐plate geometry. First, a frequency sweep from 0.1 to 10 Hz at 1% strain was performed to determine the storage modulus (*G*') and loss modulus (*G*″) of the hydrogels. Oscillatory tests were conducted with shear strains ranging from 1% to 100% at a frequency of 1 Hz. The steady shear viscosities of HME precursors were measured by varying the shear rate between 0.1 and 10 s^−1^. For surface morphology analysis, HME hydrogels were sputter‐coated with a thin gold layer under vacuum and observed using a field‐emission scanning electron microscope (N7000, Hitachi, Tokyo, Japan).

To evaluate tissue adhesive strength, LAP‐shear tests were conducted using two pieces of fresh porcine skin, each measuring 10 mm × 25 mm. A 200 µL volume of the HME precursor was evenly spread onto one skin section, and the other section was placed over it to cover the adhesive. The junction was then exposed to 365 nm UV light for 12 s to initiate crosslinking. The adhesion strengths of the samples were measured using an Instron Materials Test system (MTS Criterion 43, Instron, USA) equipped with a 50 m load cell at a speed of 10 mm/min^−1^. To assess the adhesive properties in a biological context, the HME precursor was crosslinked with rat organs and images were captured using a Nikon DXM1200F camera.

To evaluate the hemolytic activity of HME, pericardial blood was harvested from Sprague–Dawley rats and placed in 10 mL blood collection tubes containing ethylenediaminetetraacetic acid (EDTA). The blood was centrifuged at 500 × *g* for 5 min at 4 °C and the serum was carefully removed. Red blood cells (RBCs) were washed five times with an equal volume of 0.9% saline, followed by centrifugation. After washing, the RBCs were resuspended in 0.1 m PBS to a concentration of 1 × 10^8^ cells mL^−1^. Various groups containing HME hydrogels were incubated with RBC suspensions. Saline and Triton X‐100 were used as negative and positive controls, respectively. After a 120 minincubation at 37 °C, the suspension was centrifuged at 600 × *g* for 3 min. The supernatant was collected and transferred to a 96‐well plate, and optical absorbance was measured at 541 nm using a microplate reader.

The hemolysis rate was calculated using the formula

(1) $$\mathrm{Hemolysis}\mathrm{rate}[\%]=\left(A_{s}-A_{c}\right)/A_{t}\times 100\%$$

where *A*_s_ is the absorbance of the sample, *A*_c_ is the absorbance of the negative control, and *A*_t_ is the absorbance of the positive control group.

To evaluate the impact of HME concentration on cell migration, a migration assay was performed using IBIDI culture inserts (#80369, IBIDI GmbH, Germany) placed on substrates prepared with varying HME concentrations (2.0, 3.0, and 5.0 wt%). MSCs were used for the migration experiments. Cells were seeded at a density of 1 × 10^4^ cells per well onto the HME‐coated substrates. Following attachment for 4 h, nonadherent cells were gently removed by washing withPBS, and the adherent cells were cultured in Dulbecco's modified Eagle medium (DMEM) supplemented with 10% FBS and 1% penicillin–streptomycin at 37 °C in a humidified atmosphere containing 5% CO₂. To assess migration efficiency, a scratch wound assay was performed. A sterile 200 µL pipette tip was used to create a scratch across the cell monolayer after 24 h of culture on the HME substrates. The wells were washed with PBS to remove cell debris, and fresh culture medium was added. The scratch closure was monitored at 0, 12, and 24 h post scratch using a phase‐contrast microscope (Olympus IX83, Olympus Corp., Tokyo, Japan). Cell migration was quantified by measuring the wound closure area using ImageJ software (NIH, Bethesda, MD, USA).

### Microsphere Fabrication and Characterization

For BHG fabrication, a co‐flow microfluidic (#MF‐2G plus, Yongkang, China) system was used to prepare the microspheres. The process involved the following components:

1. Inner water phase: 7.5% GelMA solution with 1 × 10^7^ mL^−1^ MSCs (0.2 wt% LAP),
2. Middle water phase: 3.0 wt% HME precursors with EPCs (0.2 wt% LAP),
3. Outer oil phase: Mineral oil with 2% w/v SPAN 80.

Three phases were pumped separately into the microfluidic chip channels at adjustable flow rates to generate BHGs. The microsphere formation process was captured by a high‐speed camera (Phantom v741, Norfolk, VA, USA), and the HME cell spheres were captured under a stereomicroscope (Zeiss Stemi 305 Discovery. V12, Carl Zeiss, Germany). After removing the oil, the BHGs were implanted under dorsal skin of BALB/c nude mice, followed by UV light exposure at 365 nm for 10 s. Video S1 (Supporting Information) visually illustrates the microfluidic sphere preparation.

For microsphere degradation, the BHGs were treated with 5 U mL^−1^ hyaluronidase and 2.5 U/mL type I collagenase, and the enzyme solution was refreshed daily. At the designated time points, the samples were washed, lyophilized, and weighed. The degree of degradation was determined using the following formula

(2) $$\mathrm{Degradation}\;\left[\%\right]=\frac{W_{0}-W_{t}}{W_{0}}\times 100\%$$

where *W_t_* is the dry weight of the sample after time *t* and *W*_0_ is the initial dry weight.

For the characterization of bioengineered HME microspheres, microspheres were placed in 24‐well plates at a density of 90 spheres per well. They were cultured in a medium composed of FBS, DMEM, and mesenchymal stem cell medium (MSCM) in a 1:5:5 ratio, supplemented with 100 units mL^−1^ of penicillin–streptomycin (#P4333, Sigma–Aldrich, USA) and rho‐associated protein kinase (ROCK) inhibitor (#Y‐27632, Sigma–Aldrich, USA), and maintained at 37 °C in a 5% CO_2_ incubator. To perform RT‐qPCR, total RNA was isolated from the cell spheres using TRIzol reagent (#T9424, Sigma–Aldrich, USA). RNA was reverse transcribed into complementary DNA (cDNA) using a cDNA Reverse Transcription Kit (#RR036A, Takara, Japan). Gene expression levels were assessed using the Ultra SYBR Mixture (#RR820A, Takara, Japan) in an ABI 7900 system. Relative gene expression was calculated by the 2−ΔΔ*Ct* method. The primer sequences used for RT‐qPCR analysis are listed in Table S3 (Supporting Information).

To preparation of HD cell spheres, HD microspheres were fabricated using Perfecta3D Hanging Drop Plates (#HDP108, InSphero, USA) in accordance with the guidelines provided by the manufacturer. MSC and EPC cells (2.5 × 10^3^ and 1.25 × 10^3^, respectively) were suspended in 40 µL of culture medium and placed in individual wells of the HD plate. The cells were cultured for 48 h under standard conditions. To create Wnt3a‐loaded HD microspheres, 2 µL of Wnt3a solution was prepared and incorporated into the pre‐existing culture medium.

To prepare Wnt3a‐loaded BHG spheres, a recombinant Wnt3a protein (#92276ES76, Yeasen Biotechnology, China) was mixed with GelMA to a final concentration of 200 ng mL^−1^. This mixture served as the inner phase to create HME (Wnt3a) spheres. After formation, the BHG spheres were rinsed with PBS to remove any residual surfactant. The release kinetics of Wnt3a were evaluated by incubating the microspheres in PBS at 37 °C and sampling the supernatant at designated intervals. The concentration of Wnt3a in the supernatant was quantified using a Wnt3a ELISA Kit (#DY1324, R&D Systems, USA) following the manufacturer's protocol. The encapsulation efficiency of Wnt3a was calculated as follows

(3) $$\begin{array}{ccc} & & \mathrm{Encapsulation}\;\mathrm{efficiency}\\ & & =\frac{\mathrm{Total}\;\mathrm{wt}\;\mathrm{of}\;\mathrm{protein}\;\mathrm{encapsulated}\;\mathrm{in}\;G/\mathrm{HAD}\;\mathrm{spheres}}{\mathrm{Total}\;\mathrm{wt}\;\mathrm{of}\;\mathrm{protein}\;\mathrm{used}\;\mathrm{in}\;\mathrm{the}\;\mathrm{initial}\;\mathrm{batching}}\times 100\% \end{array}$$

### Hair Induction Assay

To evaluate the hair induction potential of the microspheres, in situ bioengineered sphere transplantation was performed using Lichti's chamber assay. A 1.0 cm diameter wound was created on dorsal skin of BALB/c nude mice. After suturing the wound, a cap was placed over it to form a minichamber, serving as the transplantation bed for the bioengineered spheres. Each group received 1450 cell spheres, which were then crosslinked to the dermis within the minichamber for 10 s. Steady pressure was applied with tapes and sterile dressings to secure the cell spheres on the skin. After 7 day transplantation, the cap was removed. The grafts were examined 14 days post‐graft using a Zeiss Discovery V8 stereomicroscope (Carl Zeiss, Germany).

### mRNA Sequencing

Total mRNA from the HD, HD (Wnt3a), HME, and HME (Wnt3a) groups was subjected to mRNA sequencing. RNA quality was verified using an Agilent 2100 Bioanalyzer (Agilent Technologies, Santa Clara, CA, USA). Quality control of the raw sequencing data was performed using the Fast QC tool. The DESeq package was used to determine genes that were differentially expressed between groups. Enrichment significance thresholds were set as a false discovery rate of <0.05 and a fold change of >2. Enrichment analysis for GO terms and KEGG pathways was conducted to identify biological processes and key signaling pathways associated with overlapped genes. GO and KEGG functional analyses were performed by OmicShare (https://www.omicshare.com/tools/) and DAVID Tools (https://david.ncifcrf.gov/). PCA and Pearson's correlation coefficient were performed using the OmicShare tools. For gene expression pattern analysis, the Short Time‐series Expression Miner (STEM) tool was employed. To explore PPIs, genes identified through STEM were analyzed using the STRING database (https://string‐db.org/) and visualized using Cytoscape software. The most significant interaction modules were determined using the Molecular Complex Detection plugin with a cutoff score of >5 in Cytoscape. Hub genes were identified using the CytoHubba plugin and ranked based on Maximal Clique Centrality in Cytoscape.

### Western Blot Analysis

Skin in the transplanted area of the nude mice was isolated and homogenized in radioimmunoprecipitation assay buffer (RIPA) buffer (#R0278, Sigma, USA) inhibitors on ice. Total protein was measured using a bicinchoninic protein assay kit (#B9643, Sigma, USA). After being blocked with 5% fat‐free dry milk in Tris‐Buffered Saline with Tween 20 (TBST), the blotting membranes were exposed to the following primary antibodies: HIF‐1α (#ab2185, Abcam, UK), p‐EGFR (#ab182618, Abcam, UK), EGFR (#ab52894, Abcam, UK), CD44 (#ab157107, Abcam, UK), STAT3 (#ab68193, Abcam, UK), TCF7 (#ab315390, Abcam, UK), *Ctnnb1* (#ab32572, Abcam, UK), LEF1 (#ab137872, Abcam, UK), Cdc42 (#ab187643, Abcam, UK), RhoA (#ab187027, Abcam, UK), IL‐6 (#ab314470, Abcam, UK), IL‐1β (#ab283818, Abcam, UK), TNF‐α (#ab183218, Abcam, UK), and GSK‐3β (#ab32391, Abcam, UK) overnight at 4 °C. After washing, the blots were incubated with the corresponding secondary antibody (#ab6721, Abcam, UK) for 1 h at room temperature (RT) and photographed using an Odyssey infrared fluorescent scanning imaging system (Odyssey CLx, LiCOR Biosciences, USA).

### Statistical Analysis

Each experiment was performed in triplicate for each sample, and the results were presented as means ± standard deviation (SD). Data analysis was performed using one‐way analysis of variance (ANOVA). GraphPad Prism software (v.8.0 for Windows) was used for Tukey's posthoc test for multiple comparisons. Statistical significance between groups was assessed using a significance threshold of *p* < 0.05.

## Conflict of Interest

The authors declare no conflict of interest.

## Author Contributions

Y.P.C., Y.H.H., and J.J.C. contributed equally to this work. Y.P.C. and J.F.H. conducted experiments, analyzed data, performed statistical analyses, and wrote the initial version of the manuscript. Y.H.H. and J.J.C. checked and analyzed the data and revised the manuscript. J.J.B., L.J.D., H.Z.Q., and C.Q. performed animal experiments. Y.H.H. and X.B.L. provided scientific support and critically reviewed the manuscript. J.J.B. commented on the manuscript and provided scientific advice.

## Supporting information



Supporting Information

Supplemental Video 1

## Data Availability

The data that support the findings of this study are available in the Supporting Information of this article.
