# Supplementary material for: Construction of Large‐Scale Bioengineered Hair Germs and In Vivo Transplantation
Source: Adv Sci (Weinh). 2025 Mar 5;12(16):2416361. doi: 10.1002/advs.202416361 (PMC12021125; doi:10.1002/advs.202416361)
Supplement: Supplementary file 1 — Supporting Information [file ADVS-12-2416361-s001.docx]

**Supporting Information**

**
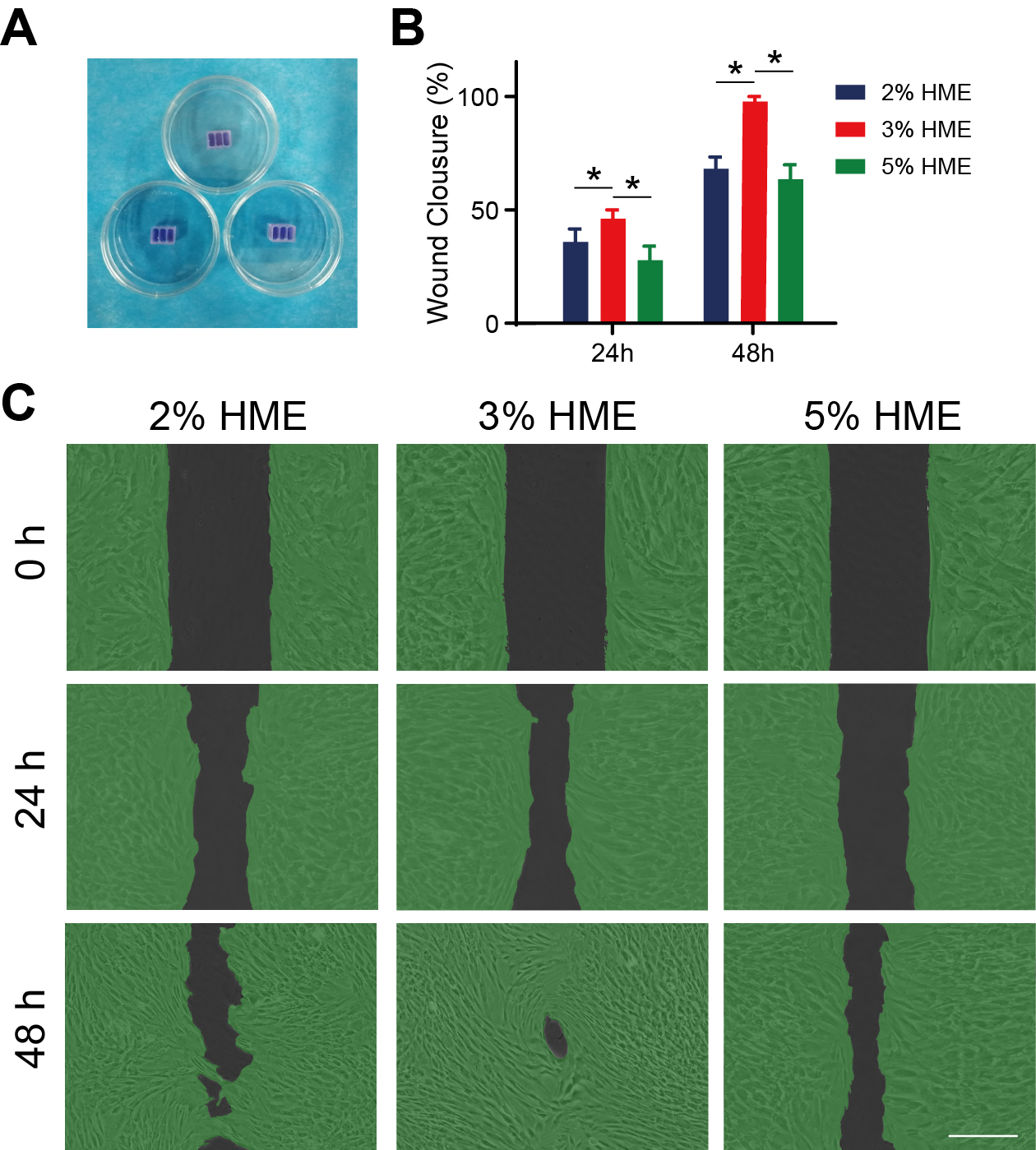
**

**Fig. S1.** MSC migration in different HME concentrations. (A) Cell Migration Model Construction Using the IBIDI Culture Inserts. (B) Wound closure fold relative to the control group at 24 and 48h, respectively, **p* < 0.01. Scale bar: 200 μm. (C) Cell migration of MSCs cultured with HME hydrogels. Relative wound closure is quantified on the right.


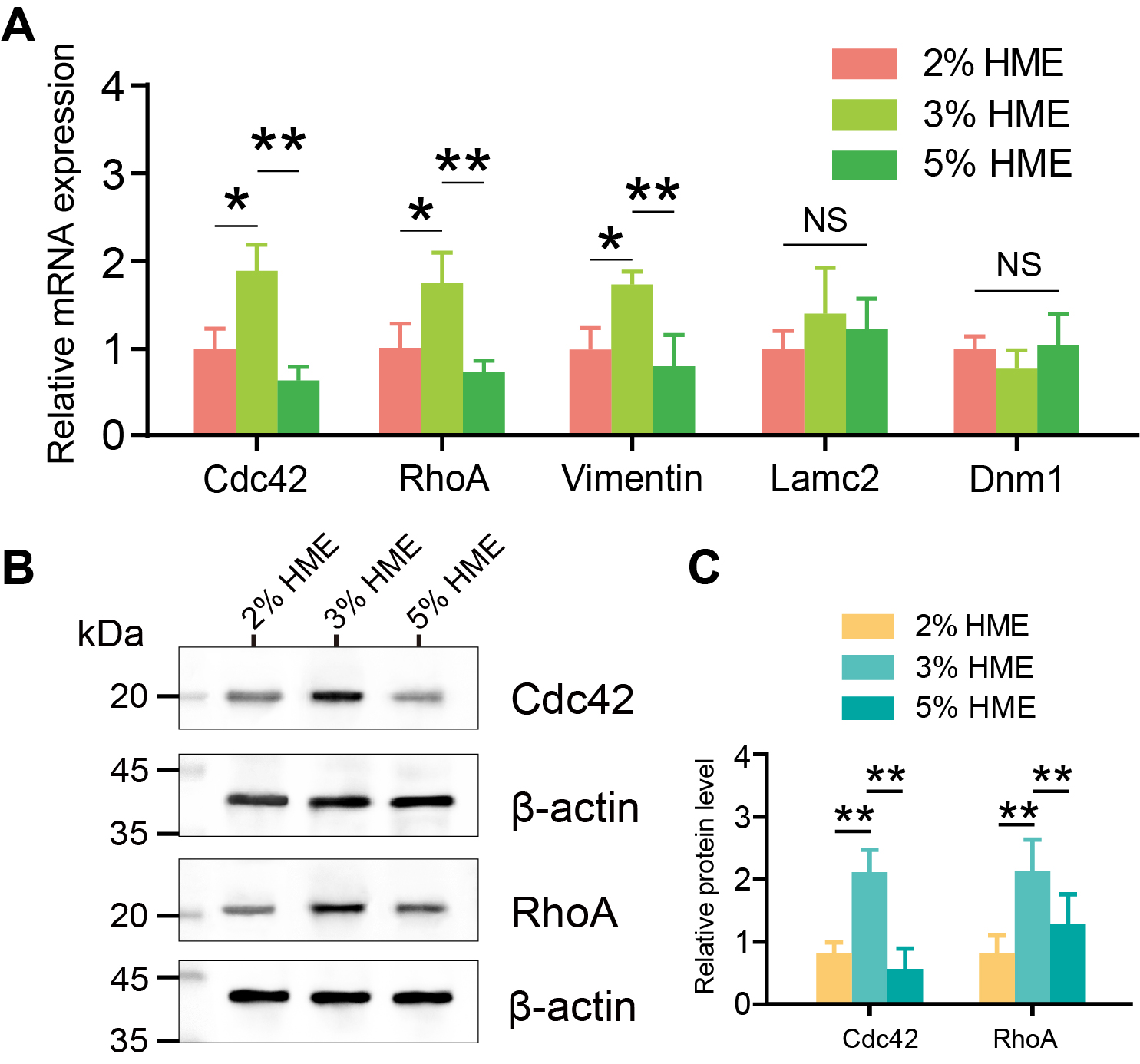


**Fig. S2**. Investigation of MSCs for immigration ability. (A) RT-qPCR of immigration-related gene expression of MSCs after culturing in HME for 3 days. (B-C) Western blot reveals changes in protein expression of immigration-related genes of MSCs after culturing in HME for 3 days. n=6. *p < 0.05, **p < 0.01. Relative mRNA and protein expression levels were normalized to β-actin.


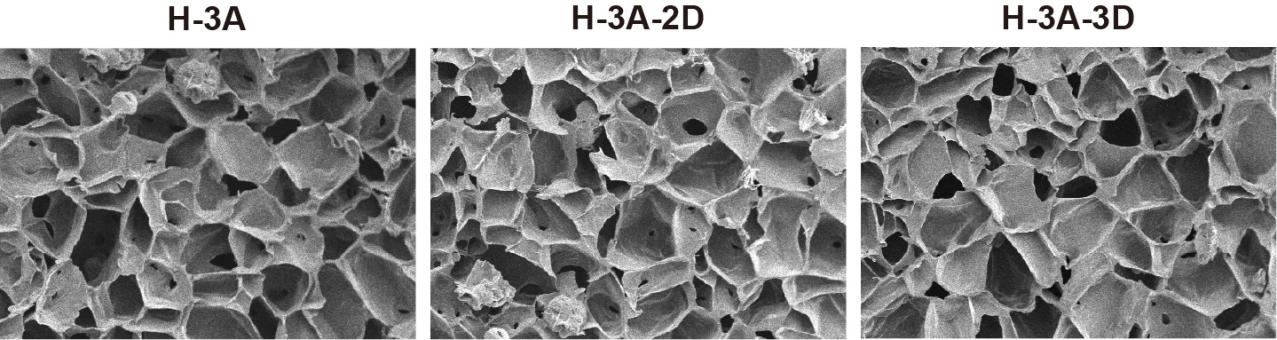


**Fig. S3.** SEM images of photo-crosslinked HME hydrogels.

**
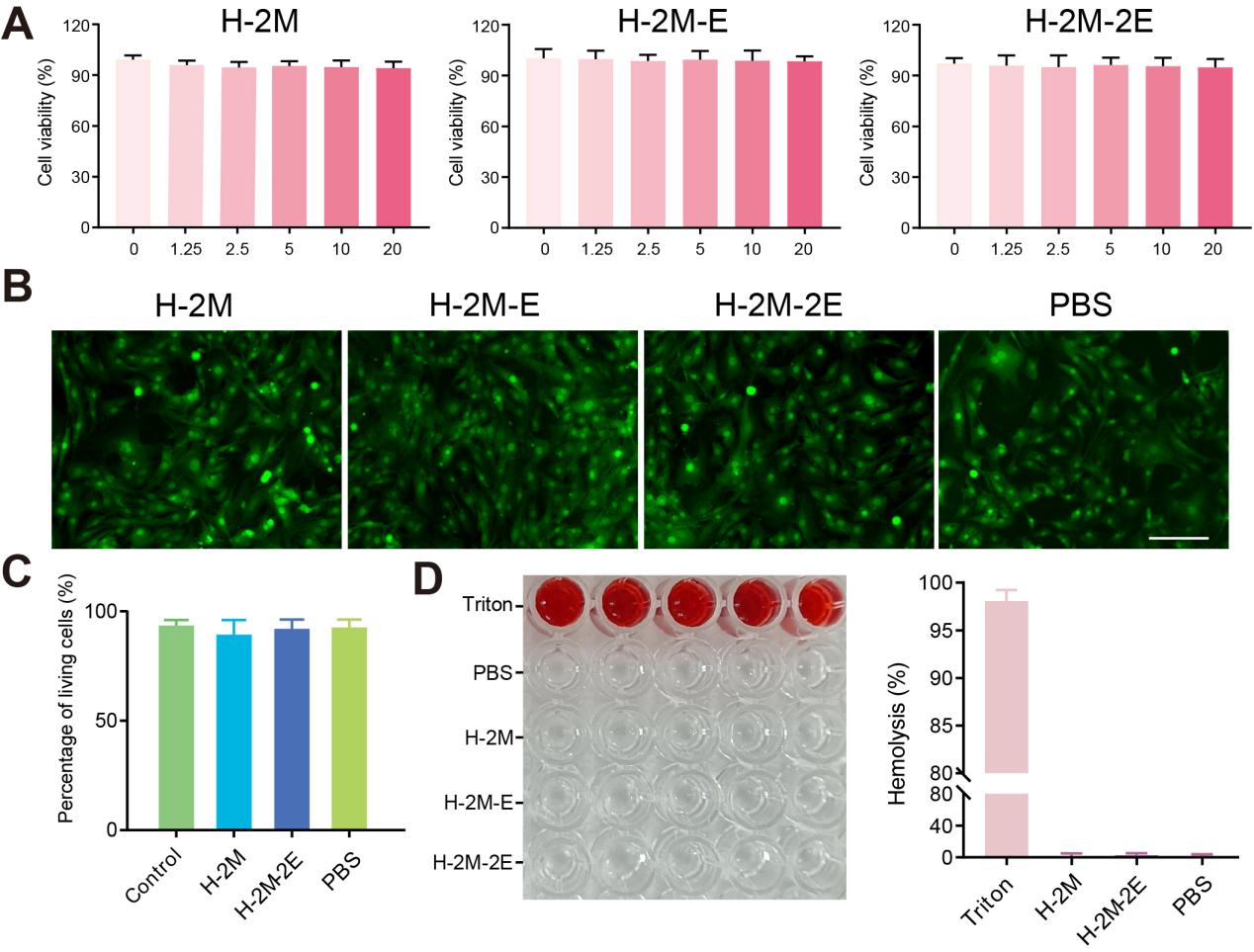
**

**Fig. S4.** Cytotoxicity and hemolysis analyses of HME hydrogels. (A-B) *In vitro* cytocompatibility of the HME hydrogel in the CCK-8 (A) and Live/Dead assay (B) of mouse mesenchymal cells after 48 h of culture. No significant difference between the each groups was observed. Scale bars = 50 μm. (C) Percentage of live cells in each group. (D) Hemolytic activity test of each group.


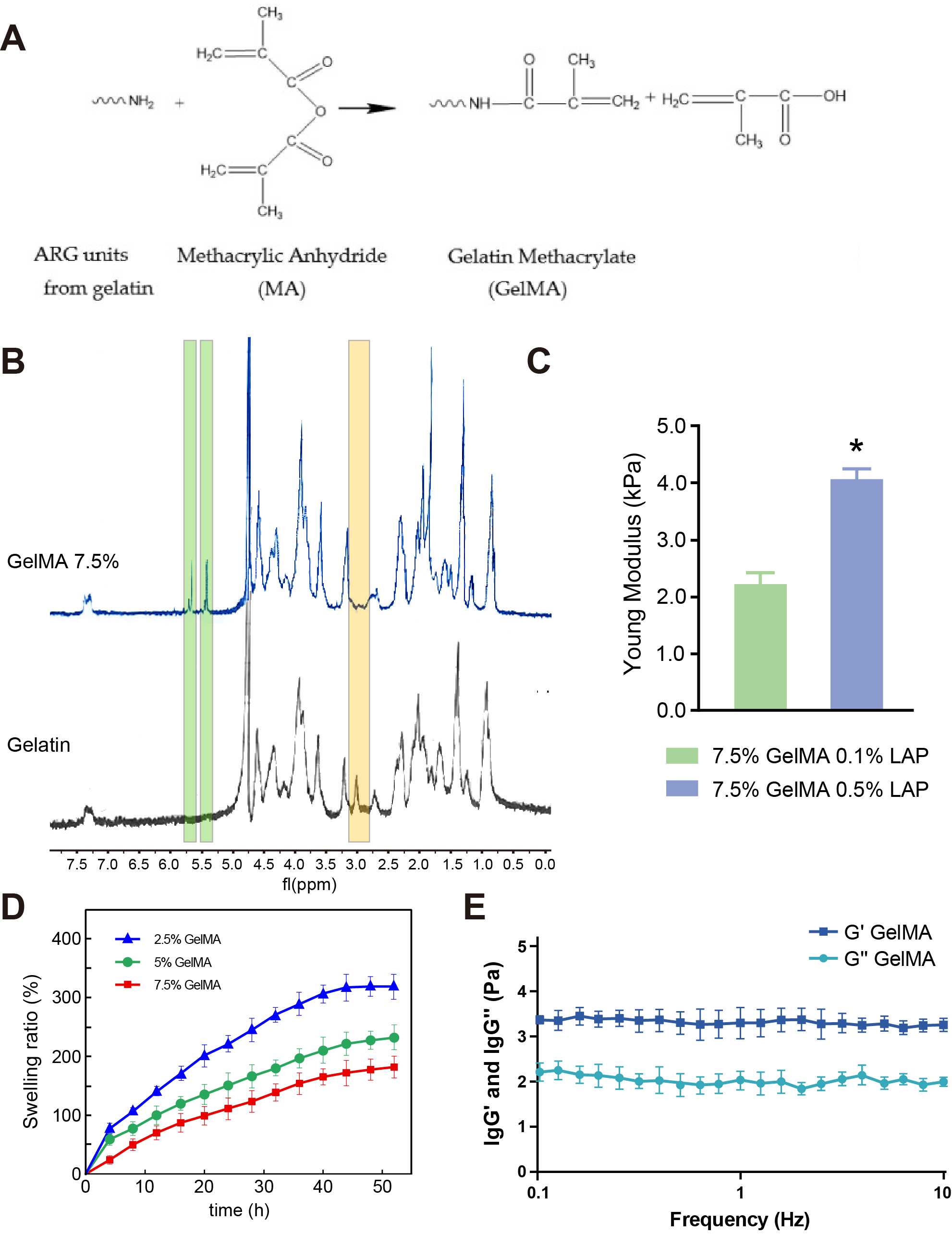
**Fig. S5.** Characterization of GelMA. (A) Methacrylation of gelatin. (B) ^1^H-NMR spectra of unmodified gelatin and GelMA. (C) Young Modulus of GelMA with different concentrations of photoinitiator. (D) Swelling rate of different concentrations of GelMA. (E) Rheological data of GelMA. Data are expressed as means ± SD. n = 9. **p* < 0.01.

**
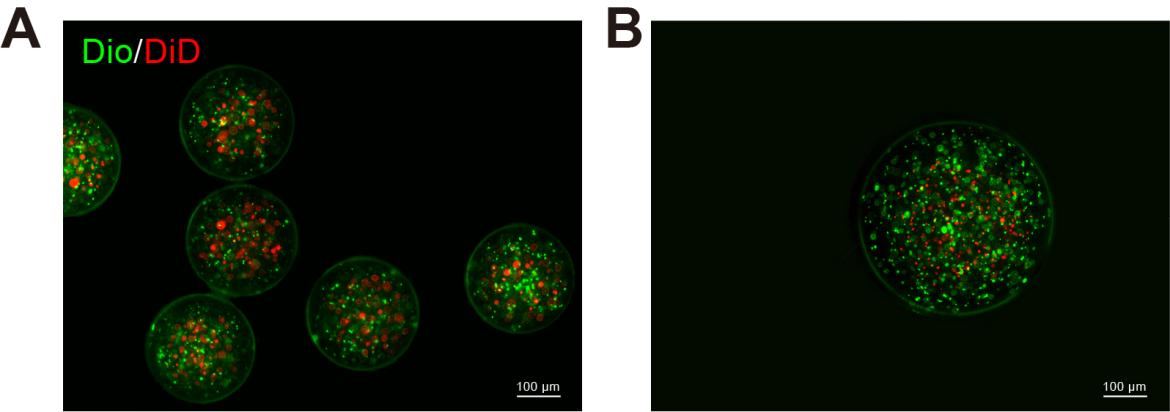
**

**Fig. S6.** Microscope image of DiD-dyeing and NucBlue-labeling MSCs within outer DiO-dyeing EPCs in microspheres after 24 h of incubation in the culture medium. Scale bar: 100 μm.

**
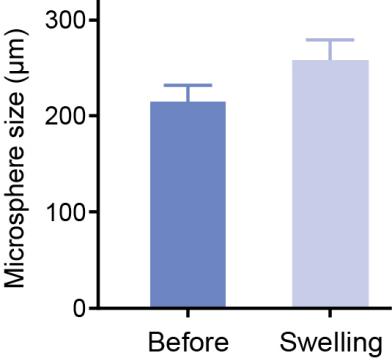
**

**Fig. S7.** Particle size distribution of HME droplets before or after swelling.

**
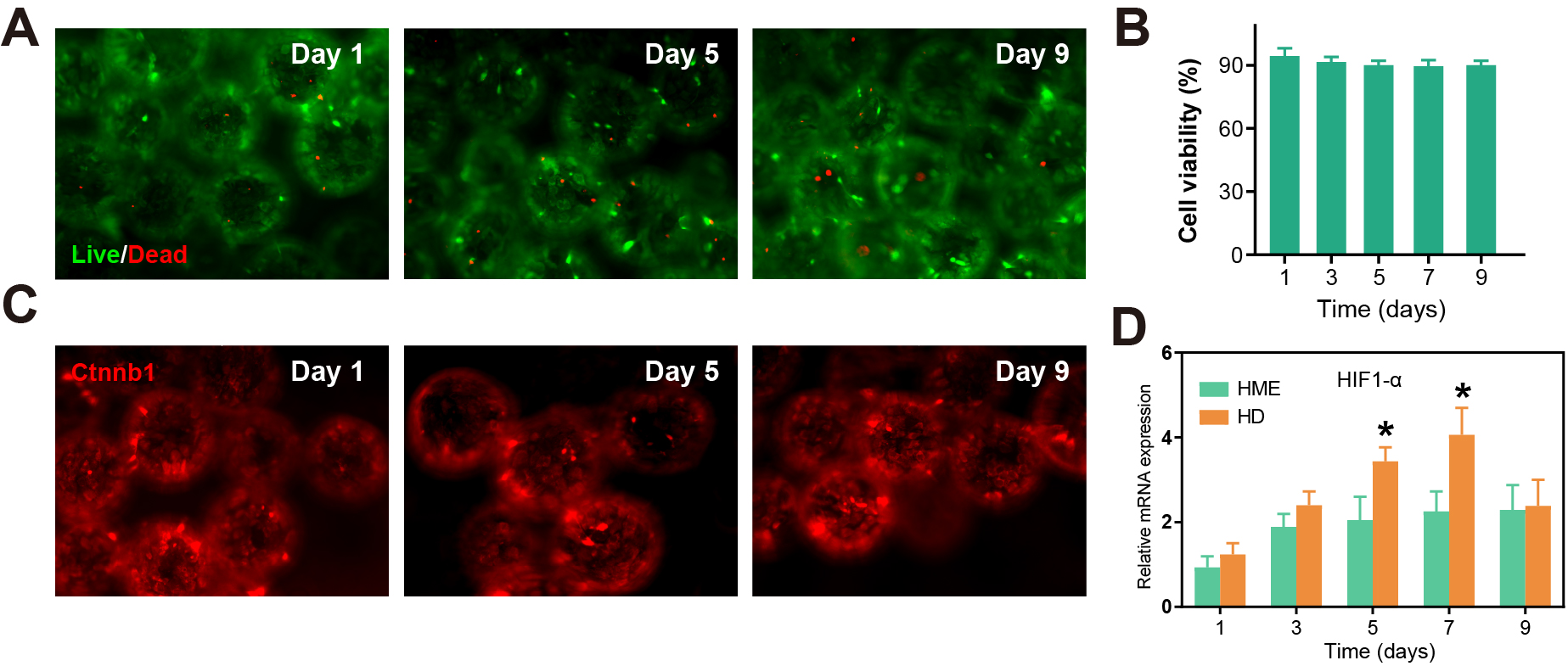
**

**Fig. S8.** Cell viability of HME microspheres. (A) Live (green)/Dead (red) cell staining of HME cell spheres. Scale bar = 100 μm. (B) Quantification of cell viability. (C) Fluorescence images showing the expression of the cell adhesion complex component Ctnnb1 (β-catenin) in HME cell spheres. (D) Relative expression of HIF-α of HME and HD cell spheres. Data are expressed as means ± SD. n = 9. **p* < 0.01.

**
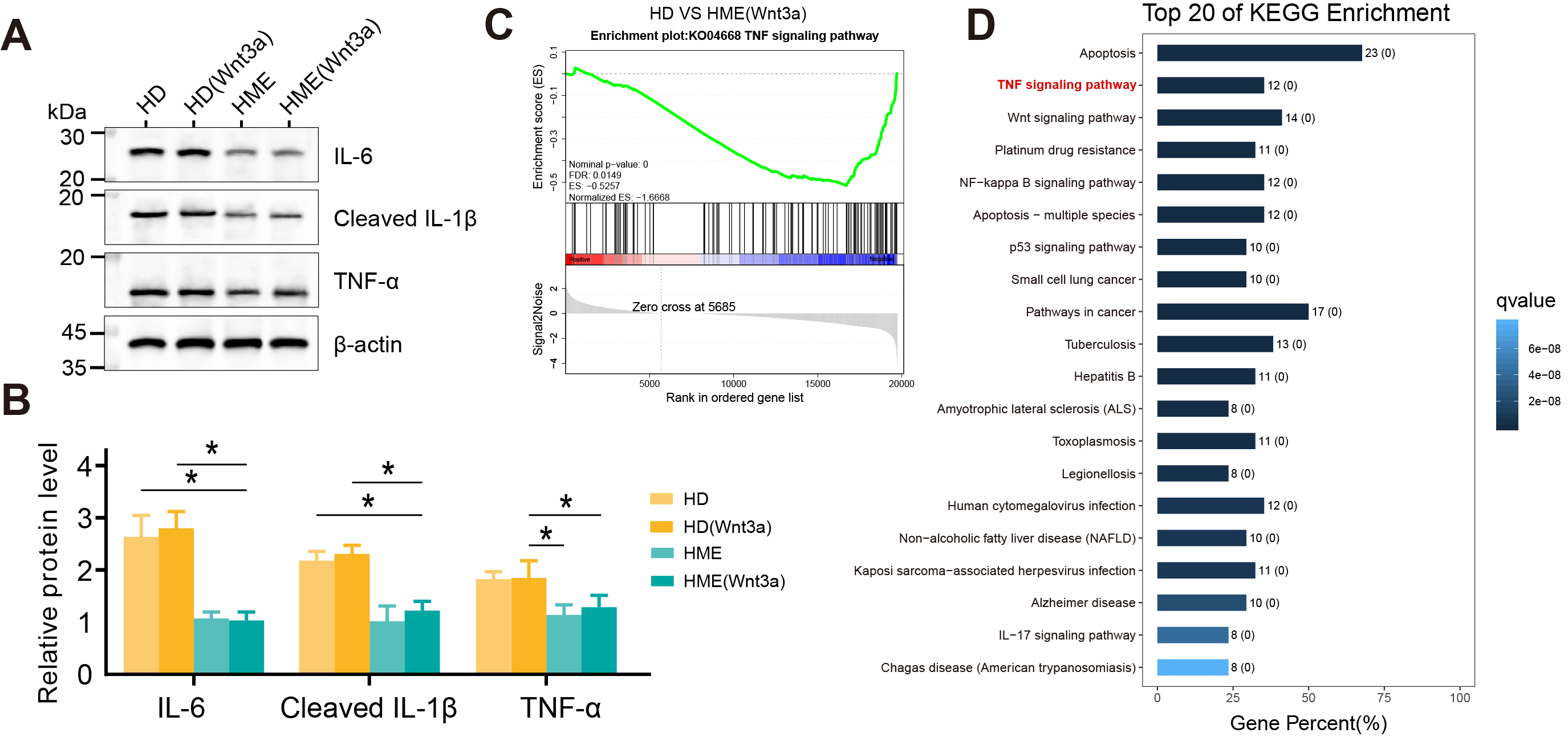
**

**Fig. S9.** HME reduces the expression of inflammation-related genes and in the skin. (A-B) Western blot analysis and corresponding quantification showing reduced protein expression levels of IL-6, IL-1β, and TNF-α in the HME group compared to the HD group. (C) GSEA (Gene Set Enrichment Analysis) identifies significantly enriched pathway. (D) KEGG enrichment analisis between HD and HME groups. Data are presented as mean ± SD. n = 6. **p* < 0.01.


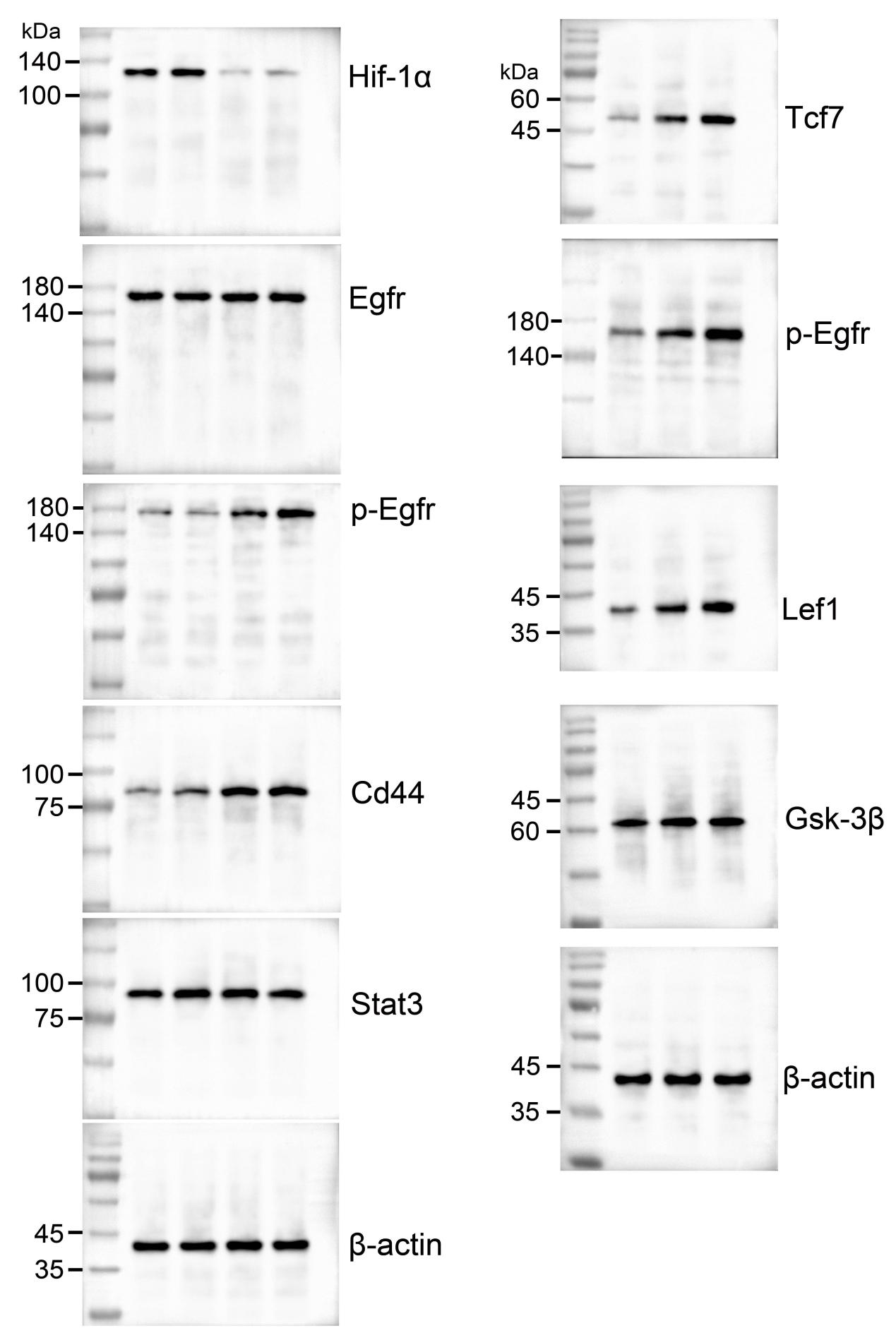
**Fig. S10.** Full-length blots/gels. The ladders used in the western blot experiments is from ColorMixed Protein Marker 180 (10-180kDa) (Catalogue number: RM19001).

**Table S1.** Comprehensive properties of constructing recent double layer structures**.**

| **Methods** | **Automation** | **Accuracy** | **Batch production per time** | **Preparation efficiency** |
| --- | --- | --- | --- | --- |
| **Microfluidic BHG**  **(This study)** | √ | high | unlimited | very high |
| **Hanging drop** | × | × | limited | low |
| **Micro-array chip** | × | medium | 96 | low |
| **Ultra low adsorption culture plate** | × | low | 96 | low |
| **3D printing double layered structure** | √ | high | <100 | medium |

**Table S2.** Reagents and Materials List.

| **Name** | **Catalog Number** | **Company** | **Country of Origin** |
| --- | --- | --- | --- |
| IBIDI Culture Inserts | #80369 | IBIDI | Germany |
| Type A porcine skin gelatin | #G1890 | Sigma-Aldrich | USA |
| Methacrylic anhydride | #760-93-0 | Sigma-Aldrich | USA |
| 2-aminoethyl methacrylate hydrochloride (AM) | #516155 | Sigma-Aldrich | USA |
| ethyl (dimethylaminopropyl) carbodiimide (EDC) | #E7750 | Sigma-Aldrich | USA |
| N-hydroxysuccinimide (NHS) | #130672 | Sigma-Aldrich | USA |
| dopamine hydrochloride | #H8502 | Sigma-Aldrich | USA |
| Foetal bovine serum (FBS) | #10270-106 | Invitrogen | USA |
| Alexa Fluor 594/405 Microscale Protein Labelling Kit | #A30008/A30007 | Invitrogen | USA |
| Live/dead reagent | #L3224/C3099 | Invitrogen | USA |
| Recombinant Wnt3a | #5036-WN-010/CF | R&D Systems | USA |
| Dispase (for cell isolation) | #D4693 | Sigma-Aldrich | USA |
| Phosphate-buffered saline (PBS) | #P3813 | Sigma-Aldrich | USA |
| Trypsin | #T4799 | Sigma-Aldrich | USA |
| Collagenase | #C0130 | Sigma-Aldrich | USA |
| Type A porcine skin gelatin | #G2500 | Sigma-Aldrich | USA |
| Dulbecco’s PBS (DPBS) | #D8537 | Invitrogen | USA |
| Methacrylic anhydride | #276685 | Sigma-Aldrich | USA |
| Sodium hydroxide | #S8045 | Sigma-Aldrich | USA |
| Hydrochloric acid | #H1758 | Sigma-Aldrich | USA |
| LAP (Photoinitiator for hydrogel) | #SE-3DP-0105 | StemEasy | China |
| Penicillin-streptomycin | #P4333 | Sigma-Aldrich | USA |
| ROCK inhibitor | #Y-27632 | Sigma-Aldrich | USA |
| TRIzol reagent | #T9424 | Sigma-Aldrich | USA |
| cDNA Reverse Transcription Kit | #RR036A | Takara | Japan |
| Ultra SYBR Mixture | #RR820A | Takara | Japan |
| Perfecta3D Hanging Drop Plates | #HDP108 | InSphero | USA |
| Recombinant Wnt3a Protein | #92276ES76 | Yeasen Biotechnology | China |
| Wnt3a ELISA Kit | #DY1324 | R&D Systems | USA |
| RIPA buffer | #R0278 | Sigma-Aldrich | USA |
| Bicinchoninic protein assay kit | #B9643 | Sigma-Aldrich | USA |
| HIF-1α antibody | #ab2185 | Abcam | UK |
| p-EGFR antibody | #ab182618 | Abcam | UK |
| EGFR antibody | #ab52894 | Abcam | UK |
| CD44 antibody | #ab157107 | Abcam | UK |
| STAT3 antibody | #ab68193 | Abcam | UK |
| TCF7 antibody | #ab315390 | Abcam | UK |
| CTNNB1 antibody | #ab32572 | Abcam | UK |
| LEF1 antibody | #ab137872 | Abcam | UK |
| GSK-3β antibody | #ab32391 | Abcam | UK |
| Secondary antibody | #ab6721 | Abcam | UK |

**Table S3.** Sequences of primers used in RT-qPCR

| Gene | Forward (5'→ 3') | Reverse (5' → 3') |
| --- | --- | --- |
| HIF-1α | GGGGAGGACGATGAACATCAA | GGGTGGTTTCTTGTACCCACA |
| Sox2 | GCGGAGTGGAAACTTTTGTCCT | CGGGAAGCGTGTACTTATCCTT |
| Angpt1 | ATCCCGACTTGAAATACAACTGC | CTGGATGATGAATGTCTGACGAG |
| β-catenin | CCCAGTCCTTCACGCAAGAG | CATCTAGCGTCTCAGGGAACA |
| Wnt3a | CTCCTCTCGGATACCTCTTAGTG | CCAAGGACCACCAGATCGG |
| Lef1 | TGTTTATCCCATCACGGGTGG | CATGGAAGTGTCGCCTGACAG |
| Angpt2 | CAGCCACGGTCAACAACTC | CTTCTTTACGGATAGCAACCGAG |
| Il1b | GAAATGCCACCTTTTGACAGTG | TGGATGCTCTCATCAGGACAG |
| Nudt6 | GCTCAGAAGGACGGATAGCTG | CAGTGTTGAGGAATGGGGTTTT |
| Wdr95 | ATGCTGCTGAGTGTATCGAGG | TTTTCTTCAAAGGCGACGTGT |
| Ddit3 | AAGCCTGGTATGAGGATCTGC | TTCCTGGGGATGAGATATAGGTG |
| Dsp | GACCTGCGCTACGAGATGAC | GGAGTGGGAGTAGAACGTCCT |
| Foxn1 | CTGCTCGTCGTTTGTGCCT | TGCCTCTTGTAGGGGTGGAAA |
| Enc1 | CTGTTTCATAAGTCCTCCTACGC | GTGGATGGAATTGTCGAAGTTCA |
| Pnpla3 | ATGGACCTCGTGCGGAAAG | CCTGGAGCCCGTCTCTGAT |
| Dpp4 | CCGTGGAAGGTTCTTCTGGG | GCTGCCGCTTCATCTTTGC |
| Angpt1 | ATCCCGACTTGAAATACAACTGC | CTGGATGATGAATGTCTGACGAG |
| Tek | TCTGATGCCGAAACATCCCTC | CCTCCAGTGGATCTTGGTGC |
| P2ry1 | GAGGTGCCTTGGTCGGTTG | CGGCAGGTAGTAGAACTGGAA |
| P2rx1 | CGGATGGTGCTGGTACGAAA | CACTGACACACTGCTGATAAGG |
| Cdc42 | CCCATCGGAATATGTACCAACTG | CCAAGAGTGTATGGCTCTCCAC |
| RhoA | GAAACTGGTGATTGTTGGTGATG | ACCGTGGGCACATAGACCT |
| Vimentin | CGTCCACACGCACCTACAG | GGGGGATGAGGAATAGAGGCT |
| Lamc2 | TTGCCTCAACTGCAATGACAA | TCTCGATGTCGGTAAAACCCC |
| Dnm1 | AATATGCCGAGTTCCTGCACT | GTCTCAGCCTCGATCTCCAG |
| IL-6 | CTGCAAGAGACTTCCATCCAG | AGTGGTATAGACAGGTCTGTTGG |
| IL-1β | GAAATGCCACCTTTTGACAGTG | TGGATGCTCTCATCAGGACAG |
| TNF-α | CAGGCGGTGCCTATGTCTC | CGATCACCCCGAAGTTCAGTAG |
| β-actin | AGCCATGTACGTAGCCATCC | GACTCCATCACAATGCCAGT |
